# Determining biocide efficacy for treating established sulfate-reducing biofilms using flow cell systems

**DOI:** 10.3389/fmicb.2025.1646177

**Published:** 2026-01-23

**Authors:** Gloria N. Okpala, Anna L. Walker, Craig Brideau, Pina Colarusso, Lisa M. Gieg

**Affiliations:** 1 Department of Biological Sciences, University of Calgary, Calgary, AB, Canada; 2 Live Cell Imaging Laboratory, Department of Physiology and Pharmacology, Cumming School of Medicine, University of Calgary, Calgary, AB, Canada

**Keywords:** 16S rRNA gene sequencing, biocide, biofilm flow cells, biofilms, sulfate—reducing bacteria

## Abstract

Sulfate-reducing microorganisms (SRM) can contribute to souring and to the corrosion of infrastructure built to support many industrial operations, including in aquatic environments. While chemicals such as biocides can effectively treat planktonic cells, less is known about biocide efficacy for treating established biofilms potentially plaguing infrastructure. We used a biofilm flow cell system to examine the efficacy of sodium nitroprusside (SNP, a nitrosating compound proposed as a “green” biocide) and alkyl dimethyl benzyl ammonium chloride (ADBAC), a membrane-disrupting biocide used across many sectors, to mitigate existing SRM biofilms. Biofilms were treated with various amounts of SNP (15–750 ppm) or ADBAC (25–500 ppm) for 10–14 h. Biofilm responses were tracked by measuring sulfide concentrations and were also analyzed for microbial community composition and by microscopy. Planktonic SRM cultures were inhibited by 15 ppm SNP, while biofilms were only transiently inhibited by 15–750 ppm SNP. Planktonic cultures were inhibited by 10 ppm ADBAC, but 50 ppm ADBAC did not suppress sulfide production in existing biofilms. ADBAC added at 100 ppm to the biofilms showed transient inhibition while the 250 and 500 ppm treatments completely inhibited sulfidogenesis. Two-photon microscopy showed primarily viable cells following the 50 ppm ADBAC treatments, a mix of viable and non-viable cells following the 100 ppm ADBAC treatment, and non-viable cells following the 250 and 500 ppm ADBAC treatments, confirmed by quantitative analysis of the images. 16S rRNA gene sequencing showed the prevalence of *Desulfobulbus* and either *Desulfomicrobium* or *Pseudomonas* in active biofilms, with these taxa differentially persisting after many of the biocide treatments. The results revealed that higher doses of biocides are needed to effectively treat existing SRM biofilms compared to planktonic cells, and that biocide dosing may only be transiently effective. Studying the effects of chemical treatments on sessile rather than planktonic communities in aquatic environments may lead to more effective treatment strategies to mitigate problematic biofilms plaguing infrastructure degradation across many industries.

## 1 Introduction

Microorganisms inhabit diverse land and water environments across our planet with up to 80% of all microorganisms believed to exist in biofilms (Flemming and Wuertz, 2019), with economic impacts of biofilms across global sectors estimated to be $5 billion USD per year (Cámara et al., 2022). Biofilms are microbial communities that are encased in a self-produced extracellular matrix (ECM) comprised of carbohydrates, proteins, lipids, and nucleic acids and that are either attached to surfaces or exist in aggregates (Flemming et al., 2025; Flemming and Wingender, 2010; Limoli et al., 2019; Sauer et al., 2022). Such a biofilm lifestyle offers many advantages to cells living within the biofilm, including metabolic heterogeneity, easy exchange of genetic information for improved fitness, and enhanced protection of cells from many external toxins (Flemming et al., 2016). It has been well documented that compared to planktonic populations, cells within biofilms are more resistant to antibiotics, metals, and other substances such as biocides (Ciofu et al., 2022; Flemming et al., 2025; Liu et al., 2024).

Although biofilms have been primarily studied in a human health context, biofilms can also be found attached to industrially relevant surfaces and can be beneficial or detrimental. For example, biofilm aggregates play critical roles in the proper functioning of water and wastewater treatment systems (Philipp et al., 2024). However, in other industrial environments such as fluid transporting pipelines, filter systems, or on marine structures, biofilms can form on the surfaces, leading to fouling that can block fluid flow or lead to the corrosion of infrastructure. Corrosion of materials due to the activity of biofilm-associated microorganisms is termed microbiologically influenced corrosion (MIC). Many types of microorganisms are known to be associated with MIC through various mechanisms, detailed in several recent review articles (Knisz et al., 2023; Puentes-Cala et al., 2022; Xu et al., 2023). However, sulfate-reducing microorganisms (SRM) are the best studied group of microorganisms related to metal corrosion (Enning and Garrelfs, 2014; Knisz et al., 2023). These organisms respire by converting sulfate to sulfide, which can then chemically react with Fe^2+^ released from carbon steel in aqueous settings, producing corrosive FeS (Enning and Garrelfs, 2014).

Biofilms can readily form in marine environments on both natural materials such as ocean flora and fauna, and on man-made materials such as plastic debris, energy infrastructure like wind farm installations (monopiles) and crude oil production platforms, docking materials in harbors, seawater cooling systems in coastal power stations, and in the hulls of ships leading to infrastructure damage or fouling (Flemming and Wuertz, 2019; Ma et al., 2020; Rubio et al., 2015; Tuck et al., 2022; Vigneron et al., 2016; Wade et al., 2011). Notably, with seawater containing 25–30 mM sulfate, biofilms containing SRM can form on surfaces, potentially leading to MIC. Thus, understanding biofilm eradication in marine systems is warranted to help prevent the destruction of industrially important infrastructure (Rubio et al., 2015; Vigneron et al., 2016).

**Figure 1:**
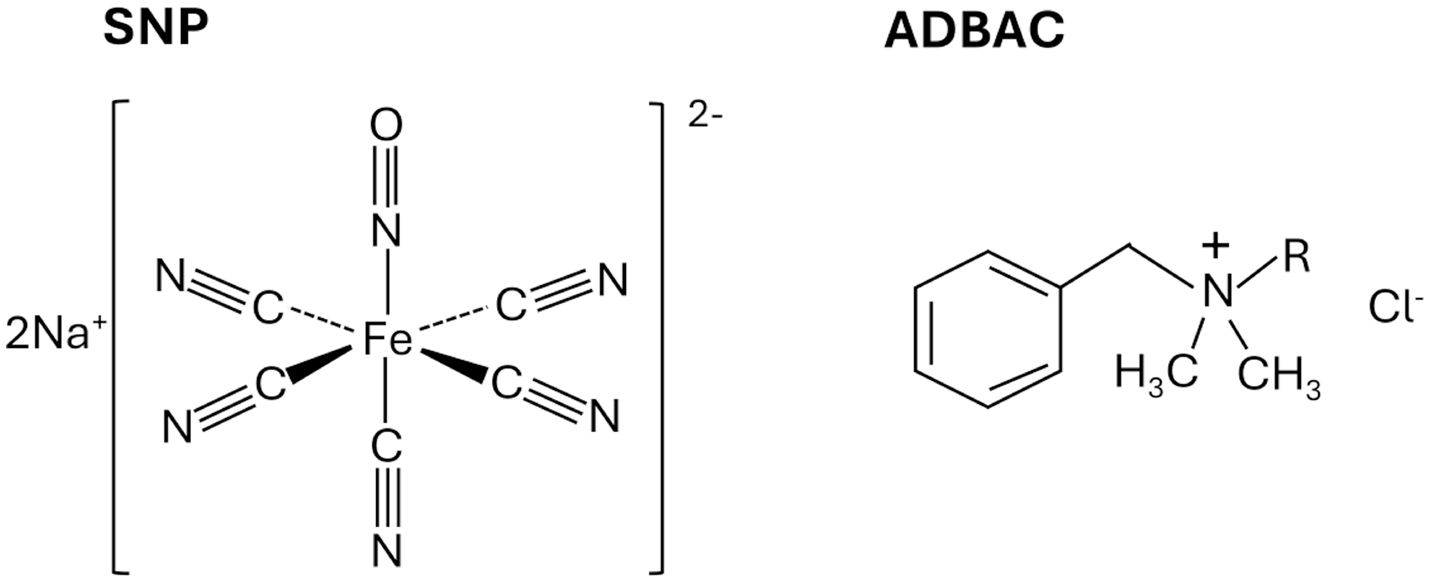
Structures of sodium nitroprusside (SNP) and alkyldimethylbenzylammonium chloride (ADBAC), the chemicals used in this study to treat sulfate-reducing biofilms. The R group in ADBAC represents an alkyl group that can range from C_8−_C_18_.

Detrimental biofilms growing in different environments can be difficult to remove given the robustness of their ECM (Flemming et al., 2025). To combat harmful biofilms, industries often apply chemical agents called biocides to help eradicate or minimize detrimental microbial activity or growth. There are many different types of biocides that target different parts of microbial cells such as membranes or protein synthesis machinery (Morris and Van Der Kraan, 2017). Some commonly used non-oxidizing biocides include glutaraldehyde, THPS, and quaternary ammonium compounds (QACs) such as alkyldimethylbenzylammonium chloride (ADBAC) (Morris and Van Der Kraan, 2017; Shi et al., 2023) (Figure 1). QACs are widely used in many different sectors (clinical, agriculture, pharmaceutical, energy), with their main mode of action being membrane disruption leading to cell lysis (Boyce, 2023; Grobe et al., 2002; Ioannou et al., 2007; Jennings et al., 2015; Obłąk et al., 2021). However, effects of QACs and other biocides on existing biofilms are poorly understood. Sodium nitroprusside (SNP; Figure 1), a vasodilator used in human medicine, has been reported to be effective against biofilms of several bacteria such as *Pseudomonas aeruginosa* and *Bacillus subtilis* (Barnes et al., 2013; Moore et al., 2004) and most recently was shown to also inhibit planktonic sulfate-reducing activity in laboratory cultures when added at low doses (Fida et al., 2018). For SNP, the mode of action is believed to be through the release of NO (nitric oxide), producing reactive nitrogen species that interact with cellular components such as lipids and DNA, resulting in a disruption of their cellular function and ultimate cell death. SNP can also chemically react with sulfide if present in a system (Quiroga et al., 2011), potentially reducing souring. The effectiveness of SNP to inhibit planktonic SRM activity led to a previous proposal that this compound could serve as a potential new “green” biocide given its safety for use in human medicine and thus having minimal toxicity to those handling and applying this compound in industrial systems (Fida et al., 2018). However, its efficacy on SRM biofilms is not known.

Historically, determining the effective dose of a given biocide was determined using planktonic cultures. However, many recent studies testing the effects of antimicrobials such as antibiotics on clinically relevant bacteria (such as *Staphylococcus aureus*, *Escherichia coli*, *Pseudomonas aeruginosa*) showed that increased dosages are required for effectively treating biofilms and that determining doses effective for biofilms is paramount (Cate et al., 2024; Ciofu et al., 2022; Grobe et al., 2002; Ioannou et al., 2007; Obłąk et al., 2021). In non-clinical environments like marine systems or industrial operations, similar principles presumably apply, but fewer studies have reported the effects of antimicrobials on already established biofilms in these environments.

In this study, we aimed to determine the effectiveness of SNP and ADBAC treatments on existing sulfate-reducing biofilms that were established using a flow cell system. Building on the effectiveness of SNP in inhibiting planktonic sulfide-producing bacteria (Fida et al., 2018), we aimed to further examine this potential “green” biocide for its efficacy on existing sulfide-producing biofilms. We also carried out parallel studies with a QAC biocide (ADBAC) already widely used across many sectors as the impacts of such biocides on existing biofilms is also poorly understood. The use of a flow cell system allowed for the application of biocides at different concentrations and the observed effects on sulfate-reducing activity, along with an evaluation of biofilm activity once biocide application was removed. We monitored biocide effects on sulfate-reducing activity (e.g., sulfide production) rather than impacts on cell numbers (e.g., cell kill rate) and augmented the impacts of various biocide concentrations by evaluating biocide penetration into biofilms using confocal or two photon microscopy. We also compared biocide dose effects on corresponding planktonic cultures. Finally, we determined microbial taxa within the biofilms in the absence of treatment and in the presence of different biocide doses to determine whether biocide addition altered the microbial community compositions of biofilms.

## 2 Methods

### 2.1 Source of sulfate-reducing enrichment culture

Pipeline pigging sludge collected during a routine cleaning of a transmission pipeline transporting crude oil served as an inoculum to enrich for SRM (Gieg et al., 2020). Approximately 1 g of the pigging sludge was weighed into sterile serum bottles, and 50 mL of freshly prepared anoxic and sterile CSBK (Coleville Synthetic Brine medium K; Callbeck et al., 2013) medium containing 10 mM sulfate (added as sodium sulfate) and 3 mM volatile fatty acids (VFA: acetate, propionate and butyrate) were added. The serum bottles were stoppered with butyl rubber stoppers and sealed with aluminum crimps. The bottles were incubated at 30 °C, and sulfide concentrations were monitored to confirm sulfate-reducing activity. After a few transfers, propionate (13.3 mM, added as sodium propionate) was added as the sole carbon source (as separate experiments showed that this substrate supported faster growth than the VFA mixture; not shown). The culture was repeatedly transferred in CSBK medium containing 10 mM sulfate and 13.3 mM propionate to maintain its sulfate-reducing activity for use in all the experiments described here. The SRM enrichment culture was always freshly transferred and monitored for sulfate-reducing activity before transferring a second time for inoculation of flow cell experiments. Following the initial enrichment, the sulfate-reducing culture was maintained at room temperature (20–22 °C) and all experiments described herein were performed at this temperature.

### 2.2 Sulfide and sulfate measurements

The activity of planktonic and biofilm SRM cultures were determined by monitoring dissolved sulfide using a spectrophotometric sulfide assay (Cline, 1969). In some cases, sulfate concentrations were also measured by ion chromatography using procedures described by Okpala et al. (2017). Planktonic cultures were aseptically sampled using sterile, N_2_-flushed needles and syringes with 10 μL of sampled fluid immediately added to 200 μL of 0.1 M zinc acetate to trap dissolved sulfide. During the operation of the flow cell experiments, direct sampling from the effluent tubing occurred daily during medium introduction, before the end of biocide treatment, and during medium re-introduction. To limit volatilization of sulfide into the atmosphere, effluent tubing was placed into a microcentrifuge tube containing 5 μL of 1 M ZnCl_2_ to trap sulfide. A minimum of 0.5 mL effluent was collected. Sulfide concentrations were measured immediately after this sample collection.

### 2.3 ATP assay

To estimate the active number of cells in the SRM planktonic culture inoculated into the flow cell system, intracellular ATP concentrations were measured using a commercially available ATP test kit according to the manufacturer’s instructions (LuminUltra Technologies, Fredericton, NB). Briefly, samples were filtered, followed by cell lysis to release the ATP. The ATP and luciferin-luciferase reagent were mixed and measured on a luminometer (PhotonMaster, LuminUltra Technologies, Fredericton, NB). The conversion of RLUs, the initial output from the luminometer, to final units of microbial equivalents (ME) of ATP per unit of volume (mL) was done. Volume of sample, background readings, and standard of reagents were accounted for.

### 2.4 Biocide stock solutions

Sodium nitroprusside dihydrate was obtained from Sigma-Aldrich (Oakville, ON) and was freshly prepared before each use as a 10 mM (3,000 ppm) sterile anoxic stock solution by dissolving 0.015 g SNP in 5 mL deionized water. ADBAC was obtained as a commercial product from SUEZ North America. A 10,000 ppm ADBAC stock solution was anoxically prepared by dissolving 1 g ADBAC into 98 mL N_2_-flushed water. Biocide stock solutions were then diluted to the desired concentrations into CSBK medium for use in the flow cell experiments.

### 2.5 Biocide treatment of planktonic cultures

The active SRM enrichment culture was used to determine the effects of different doses of SNP and ADBAC on planktonic sulfate-reducing cells. Sterile, anoxic CSBK medium containing 13.3 mM propionate and 10 mM sulfate was inoculated with 10% vol/vol of the enrichment culture. The sulfate and sulfide concentrations were monitored to determine the approximate mid-log phase of the culture (the point at which about half of the added sulfate has been reduced to sulfide). For the SNP tests, 20 mL of the mid-log phase culture were transferred with a N_2_-flushed syringe into sealed 60 mL sterile serum bottles containing a N_2_/CO_2_ (90/10) headspace. The planktonic cells were immediately challenged with 0, 7.5, 15, 30, 75, or 150 ppm SNP (in triplicate) based on effective doses previously reported by Fida et al. (2018). All treatments were performed in triplicate. After the treatment, the cultures were monitored for 20 days for sulfide (from each replicate bottle) and sulfate (in a single bottle for each treatment) to ascertain the impact on sulfate-reducing activity. The ADBAC dosing tests were performed similarly but were amended with 0, 10, 50, or 100 ppm ADBAC, performed in triplicate. These doses were chosen to span a similar range as those tested for SNP. Sulfide concentrations were monitored over a period of 20 days, followed by a 10% transfer of each treatment inoculated into fresh CSBK medium. Sulfide concentrations of the newly transferred treatments were monitored for an additional 10 days to determine whether any viable (active) cells remained.

### 2.6 Establishment of biofilms

#### 2.6.1 Flow cell assembly and inoculation

**Figure 2:**
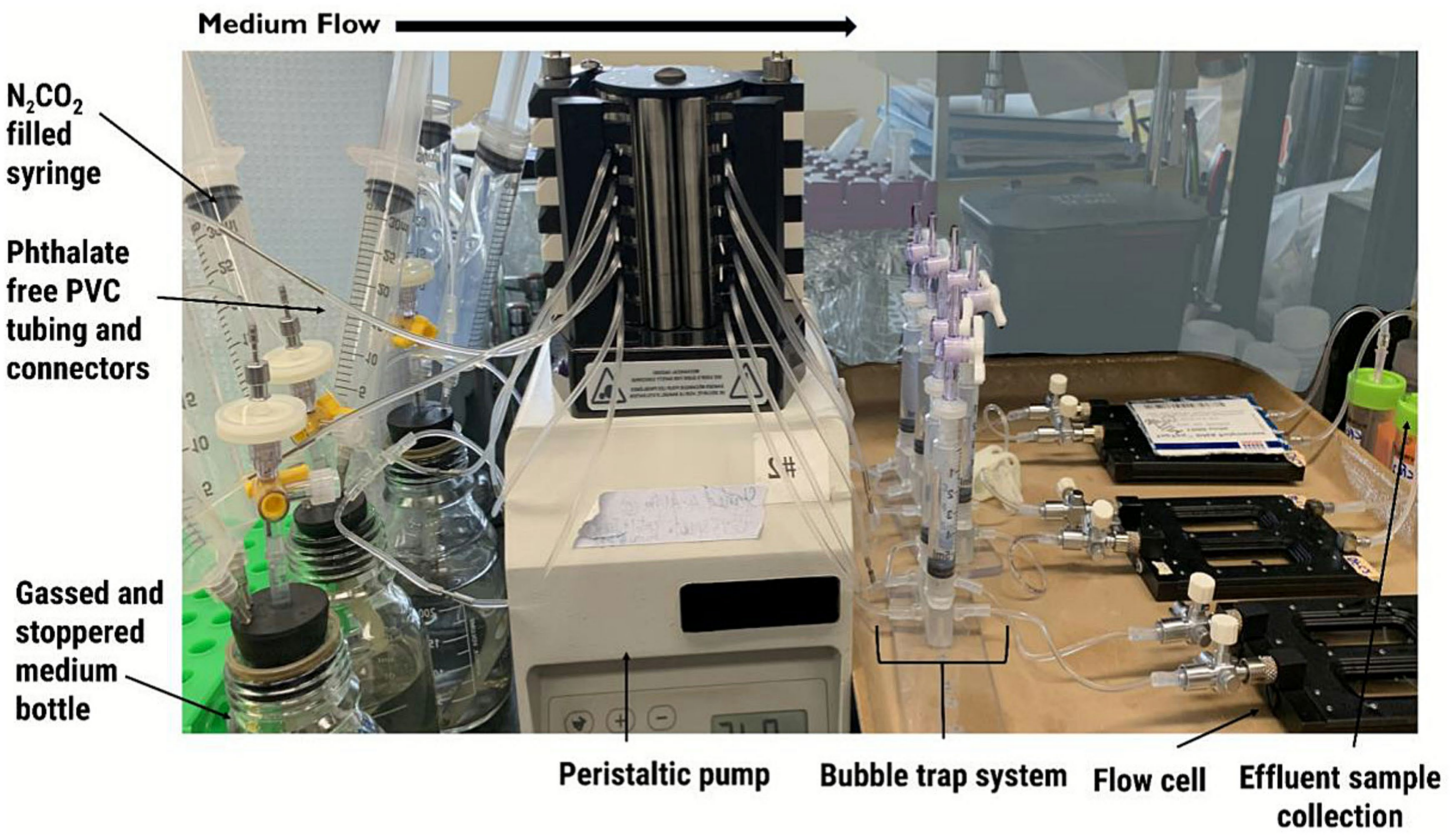
Photograph showing the set-up of the biofilm flow cell system used in this study.

To establish sulfate-reducing biofilms, FC 285-AL dual channel transmission flow cells (BioSurface Technologies Corp., Bozeman, MT) were assembled as per the manufacturer’s instructions. Note that this flow system was used for biofilm establishment, applying dosages of biocides to established biofilms for a set period of time, and allowed for biocide removal over time to assess the longer-term effects of biocide treatment on the biofilms. The flow cell chambers contain a glass slide for biofilm establishment. Figure 2 shows the overall flow cell set up consisting of the series of PVC tubing, metal connectors, three-way valves, 0.22 μm filter, pump tubing, and a bubble trap system that was used throughout the experiment. Flow cells, tubing, valves, and all media used for the flow cell experiments along with needles and other supplies were sterilized by autoclaving prior to starting the experiment. A needle and PVC tubing (Gilson F117956, 0.76 mm) was attached to the effluent end of the flow cell. To prevent the cracking of the glass slide inside the flow cell, anoxic water heated to 60 °C was added through the three-way valve at the inlet of each flow cell chamber prior to inoculation with 4 mL of the SRM enrichment culture that had just been freshly transferred (10% vol/vol) from mid-log phase. The flow cell chamber holds 2 mL, so this injected volume ensured that the chamber was filled with inoculum.

To help ensure sterility and maintenance of an anoxic environment, the three-way inlet valve was only opened after the syringe containing inoculum was attached. Both the inlet valve and effluent tubing valves were closed and sealed during the biofilm incubation and establishment period until the medium introduction began. The planktonic SRM enrichment culture used to inoculate the flow cells was monitored for sulfide production as a proxy for biofilm establishment/activity. Once approximately half of the stoichiometrically expected sulfide was produced (4–6 mM; mid-log phase) in this planktonic culture, the biofilms were also considered ready for operating as a flow system. Five flow cell units were available for biofilm experiments. For each experiment carried out, 3 to 5 “equivalent” biofilms were established from the exact same inoculum bottle that then either remained untreated or were treated with varying concentrations of biocides. Supplementary Figure 1 shows the averages in sulfide production from the 3 or 5 equivalent biofilms established for each flow cell experiment described in this study. While variation was observed across the equivalent biofilms established across all experiments and time frames for achieving active biofilms that could then be treated also varied, all biofilms showed an upward trend of sulfide production prior to biocide treatments.

Replication of data was achieved by repeating all flow cell experiments at least twice rather than running replicate concentrations at the same time. The rationale for carrying out the experiments in this way was to help ensure that equivalent, replicate biofilms (e.g., coming from the exact same inoculum bottle) were established in order make direct comparisons between the effects of different biocide concentrations. Further, the flow cell experiments needed to be run at least twice for different analyses to be carried out (e.g., microscopy, microbial community analysis, or to test the re-establishment of SRM activity following biocide treatment) as overviewed below.

The flow cell systems were initially tested by assessing the effects of SNP on established SRM biofilms using concentrations of 0, 15, or 30 ppm SNP, or with concentrations of 0, 75, or 150 ppm SNP. All these concentrations were then tested again in a new experiment, yielding similar results (described in the Results section). The SRM biofilms treated with 300 or 750 ppm SNP were also tested in two separate experiments, though sulfide production was only monitored in one of these experiments. For the experiments assessing the effects of ADBAC on existing SRM biofilms, all concentrations of ADBAC (0, 50, 100, 250, or 500 ppm) were tested in four separate flow cell experiments - two in which flow cells were opened immediately after biocide treatment, and two in which the biofilms were allowed to re-establish following medium addition. Thus, at least duplicate biofilm flow cells experiments were conducted for both biocides tested.

#### 2.6.2 Peristaltic pump set up and medium introduction

A peristaltic pump (Mini plus 3, Gilson) was used to continuously add CSBK medium into the flow cells once biofilms were established as described above. Sterile, anoxic bottles containing CSBK medium were attached to the peristaltic pump (Figure 2). A 0.22 μm filter was placed between the medium bottle and pump tubing to help minimize any particulates and ensure sterility of fluids entering the system. A flow rate of ~ 0.5 mL/h was selected for purposes of sample collection and avoidance of biofilm disruption. The pressure was regulated in the medium bottles using N_2_/CO_2_ (90/10)-gassed syringes inserted into the medium bottles. Effluent containers were used for sample collection. To monitor sulfide concentrations in the biofilms (as a measurement of sulfate-reducing activity) effluent tubing was placed into microcentrifuge tubes containing 1 M ZnCl_2_ to trap sulfide that was then measured (as described in section 2.2).

#### 2.6.3 Biocide treatment

Once the biofilms were deemed to be at mid-log phase during the continuous flow of medium, treatments with various concentrations of biocides were then initiated. Anoxic stock solutions of the desired concentrations of either SNP or ADBAC were prepared and then added to a biofilm-containing flow cell. At the time of biocide treatment, flow of medium was temporarily halted, and bubble traps were emptied of medium and filled with respective biocide concentration solution to avoid dilution of biocide. Biocides were then introduced to each biofilm for a period of 10–14 h at the same flow rate of the previous medium addition (0.5 mL/h). After this time period, the biofilms were then assessed for either re-establishment of sulfate-reducing activity, prepared and imaged using microscopy, or dismantled shortly after biocide addition to collect the biofilm for microbial community composition (this analysis could also be performed after re-establishment of sulfate-reducing activity).

For each type of assessment after biocide treatment, a different flow cell was required, thus tested conditions were performed multiple times (as described above) to obtain different measurements. For biofilms assessed for re-establishment of sulfate-reducing activity, the re-introduction of fresh CSBK medium following biocide treatment then occurred for varying times (1 to 10 days) during which sulfide concentrations were monitored. For microscopy observations following biocide treatments, biocide-free CSBK medium was allowed to flow through the biofilms for approximately 30 min, then biofilms were processed for microscopic observations as described in section 2.7.

For microbial community composition determinations, each flow cell was opened and biofilm on the glass slide was quickly collected into a sterile microcentrifuge tube using a sterile needle; this sample was then processed further as described in section 2.8.

### 2.7 Microscopy

After biocide treatment or the re-introduction of CSBK medium to biofilms, the FilmTracer LIVE/DEAD Biofilm Viability kit (Thermo Fisher Scientific, Ottawa, ON) containing the two nucleic acid stains SYTO 9 and propidium iodide (PI), was used to differentially stain “live” or “dead” (membrane-compromised) cells, respectively. The staining procedure was performed according to the manufacturer’s instructions by slowly injecting the appropriate solutions into the flow cell. Stained biofilms were then immediately analyzed using confocal or two-photon microscopy. Different microscopes and data visualization systems were used for examining SNP- and ADBAC-treated biofilms based on availability and health precautions in place when the experiments were done (the SNP work was done when restrictions were in place due to the Covid-19 pandemic; the ADBAC work was conducted after restrictions were removed).

For the SNP-treated biofilm experiments carried out during the Covid restrictions, images were acquired using a Zeiss Axio Imager Z1 confocal laser scanning microscope LSM510 NLO with a 40x/0.8 NA objective (Zeiss, Inc., Germany), as described by Moo et al. (2013). The microscope used a 488 nm Argon laser to excite fluorescence, with a 520/20 green bandpass filter for Channel 0 and a 680/60 red bandpass filter for Channel 1. Z-stacks were acquired at 1-micron intervals and ImageJ (Schneider et al., 2012) was used to review and visualize the imaging data. Biofilms from the untreated (control), 300 ppm SNP, and 750 ppm SNP tests were visualized using this microscopy approach.

More recently, ADBAC-treated biofilm experiments were imaged separately on a multiphoton microscope (Bergamo 2, Thorlabs, USA) using ThorImageLS 3.2. Two-photon microscopy was used rather than standard confocal laser scanning microscopy due to its superior penetration into the thick biofilm sample (Neu et al., 2002; Vroom et al., 1999). Three random fields of view were imaged per condition (control, 50, 100, 250, and 500 ppm ADBAC). Fluorescence of SYTO 9 and PI was simultaneously excited via two-photon excitation using 920 nm from a Tiberius Ti: Saph laser (Thorlabs, USA). To separate the SYTO 9 and PI signals, the fluorescence emission was directed first to a primary dichroic (705 nm long pass), and then to a pair of GaASP photomultiplier tubes, each equipped with a bandpass filter (525/50; SYTO and 607/70; PI).

Large fields of view, corresponding to 640 by 640 μm were acquired using a 20×/1.0 NA (Olympus, Japan) objective. Z-stacks were acquired at 2-micron intervals, and images were recorded until where the fluorescence emission was weak and almost non-detectable. After acquisition, the image stacks were visualized using FIJI (Schneider et al., 2012) as well as Nikon Elements Analysis 4.60 software. Image quantification was carried out using Cell Profiler 4.28 (Stirling et al., 2021) by comparing the relative signal of the SYTO 9 and PI channels for every condition First, each fluorescence channel was segmented into foreground (positive signal corresponding to biofilm) and background using the FindPrimaryObjects module in CellProfiler. Settings applied included global Otsu using 3-class thresholding where only the highest intensity class was considered as foreground. The automated segmentation was verified by visual inspection. Objects were filtered so that only objects representing more than 3 pixels (corresponding to X by Y microns) were analyzed; this was to minimize measuring spurious fluctuations due to shot and other noise as fluorescence emission. The detected objects were then merged, and the fluorescence intensity in the SYTO 9 and PI images were measured for each image in each Z-stack. The integrated intensity, representing the total intensity signal over all the detected regions, was measured for each channel.

### 2.8 DNA extractions and 16S rRNA gene sequencing

Biofilms removed from the glass slides within each flow cell were processed for amplicon sequencing. Prior to DNA extraction, biofilm samples were centrifuged for 10 min at 14,000 g, and resulting pellets were resuspended in 1 mL medium. Samples were then split into two 0.5 mL samples - one for treatment with propidium monoazide (PMA) (Biotium, Fremont, CA, USA) and one remaining untreated (NPMA). PMA is a membrane impermeant dye, known to bind to DNA only in compromised cells, preventing its extraction, thus DNA from PMA-treated samples can be a better reflection of living, intact cells in any given sample (Nocker et al., 2007). For PMA-treated samples, 1.25 μL PMA were added in a dark room. Samples were covered in tinfoil and placed on a rocker for 10 min to allow for PMA to penetrate cells with compromised membranes. Samples were then placed under a halogen light (500 W) on a bed of ice for 10 min and rotated every 2 min to allow for crosslinking of PMA to DNA in cells with compromised membranes, preventing its amplification during PCR. PMA-treated samples were centrifuged at 5000 g for 10 min, then resuspended in 200 μL of phosphate buffer prior to DNA extraction. Samples were stored at −20 °C until DNA extraction could be performed.

Genomic DNA was extracted using the FastDNA™ SPIN Kit for Soil (MP Biomedicals) according to the manufacturer’s protocols. DNA was quantified by fluorometry (Qubit, Invitrogen). The V4-V5 region of the 16S rRNA gene was amplified using a 2-step PCR protocol as described by Rachel and Gieg (2020). Amplicons were further processed and sequenced on an Illumina MiSeq (v2 300 cycle) at the Centre for Health Genomics and Informatics (University of Calgary, Canada). Resulting sequences (raw reads) were processed through a series of quality control steps to remove chimeras, etc. through a DADA2 pipeline in R (Callahan et al., 2016). The R code/pipeline for sequence data processing and visualization can be found at.^1^ Supplementary Table 1 shows the number of raw reads and quality-controlled reads obtained for the sequencing results shown in this study. Quality reads were assigned as amplicon sequence variants (ASVs) to the lowest taxonomic level possible against the SIVLA 138. 2 database (Quast et al., 2012), and ASV plots were created using graphing functions in R studio. Amplicon sequencing data is available in GenBank (NCBI) under BioProject accession number PRJNA1284394.

## 3 Results

### 3.1 Growth and composition of enriched SRM culture used to create biofilms

A sulfate-reducing enrichment culture derived from pipeline pigging solids was used as the source of inoculum for all biofilm experiments. The culture was regularly maintained on propionate as the carbon and energy source and sulfate as the electron acceptor. Prior to each use as an inoculum for a biofilm experiment, the culture was transferred into fresh medium, and sulfide was monitored to ensure that it was active before it was transferred again during mid-log phase (when 4–6 mM sulfide was produced) to serve as the starting culture for inoculating the biofilms. We used the ATP assay to estimate the number of active cells being transferred and used for biofilm inoculation when sulfide values were at 4–6 mM, which typically took 5 to 7 days of incubation. Supplementary Figure 2 shows that during mid-log phase (e.g., at 6 days), the culture contained an average of 3.5 × 10^7^ active cells per mL. As the flow cells were inoculated with 2 mL of culture, all flow cells were inoculated with approximately 7 × 10^7^ cells for biofilm establishment.

The SRM planktonic enrichment used as the inoculum was sequenced twice (in late 2020 and early 2023), showing that the planktonic community was comprised of *Desulfobulbus* as the most abundant sulfate-reducer, with a lower relative abundance of *Desulfomicrobium* (Supplementary Figure 3). *Desulfobulbus* is a known propionate-utilizing sulfate-reducing bacterium (El Houari et al., 2017), aligning with the maintenance of this culture on propionate as the carbon and energy source. *Desulfobulbus* sp. are known to incompletely oxidize propionate to acetate and CO_2_ that can be used by other taxa in communities (Ozuolmez et al., 2020). Fermentative bacteria such as *Sphaerochaeta* and *Proteiniphilum*, along with the acetogen *Acetobacterium* also consistently made up the community. Changes in some of the dominant taxa in the culture showed that the community shifted over time as it was transferred and maintained over 3 years on propionate and sulfate. Regardless of these community shifts, the culture continued to demonstrate sulfate-reducing activity so it could reliably be used to establish sulfidogenic biofilms over the time that this research was conducted.

### 3.2 Evaluation of the efficacy of SNP on a planktonic SRM culture

A previous study reported that SNP added at doses as low as 15 to 30 ppm (depending on cell biomass) was effective in preventing sulfidogenic activity using a mixed SRM planktonic culture enriched from oilfield produced waters (Fida et al., 2018). This finding was confirmed in the present study using a different SRM enrichment obtained from pipeline pigging sludge, wherein SNP added at concentrations of 7.5 ppm or higher inhibited sulfide production and sulfate consumption compared to an untreated control (Supplementary Figure 4).

### 3.3 Evaluation of the efficacy of SNP to treat sulfate-reducing biofilms

**Figure 3:**
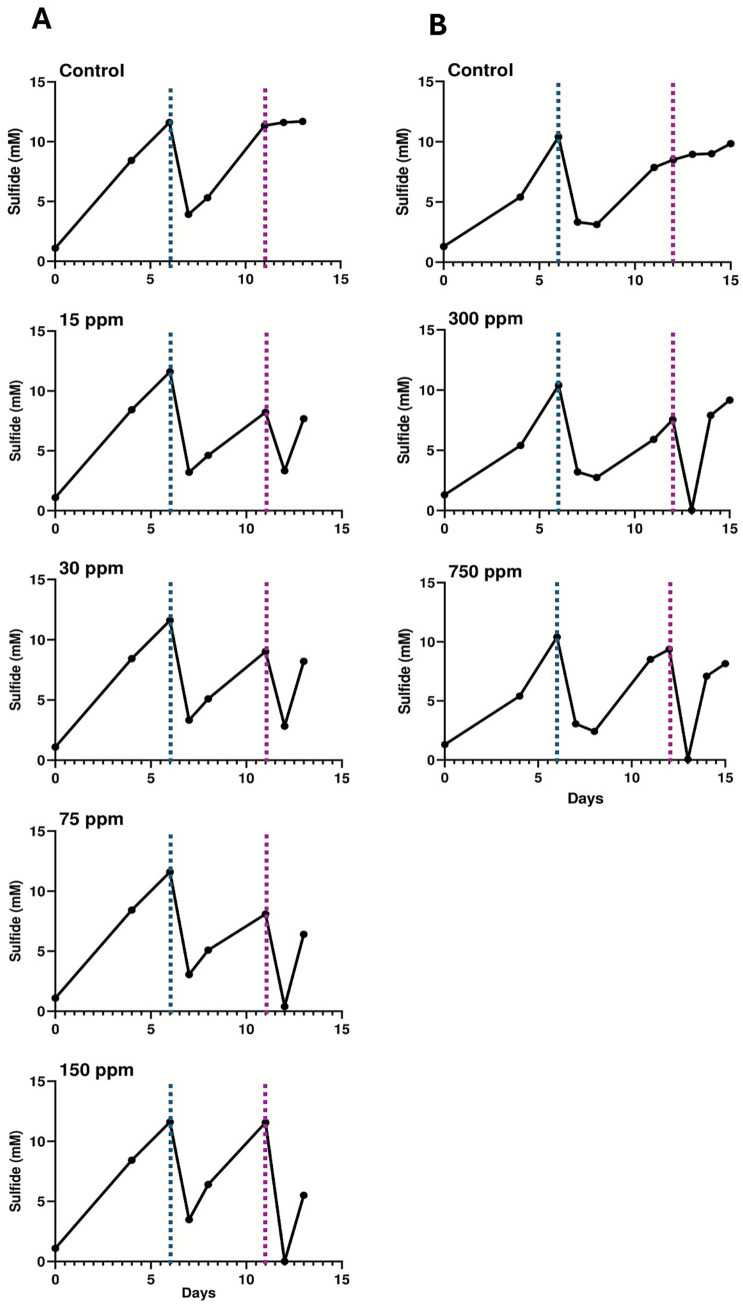
Sulfide production from biofilm flow cells treated with **(A)** lower concentrations of SNP (15–150 ppm), or **(B)** higher concentrations of SNP (300, 750 ppm). These data were generated in two different experiments, with each having a corresponding untreated control biofilm. The first phase of each experiment consisted of biofilm establishment under static conditions after which medium flow through the biofilm was initiated (as indicated by the blue lines). After sulfide production was evident during flow conditions, SNP was added to the biofilms (at the point indicated by the red lines) for 10 h. The results of replicate experiments for the 15–150 ppm SNP-treated experiments are shown in Supplementary Figure 5.

**Figure 4:**
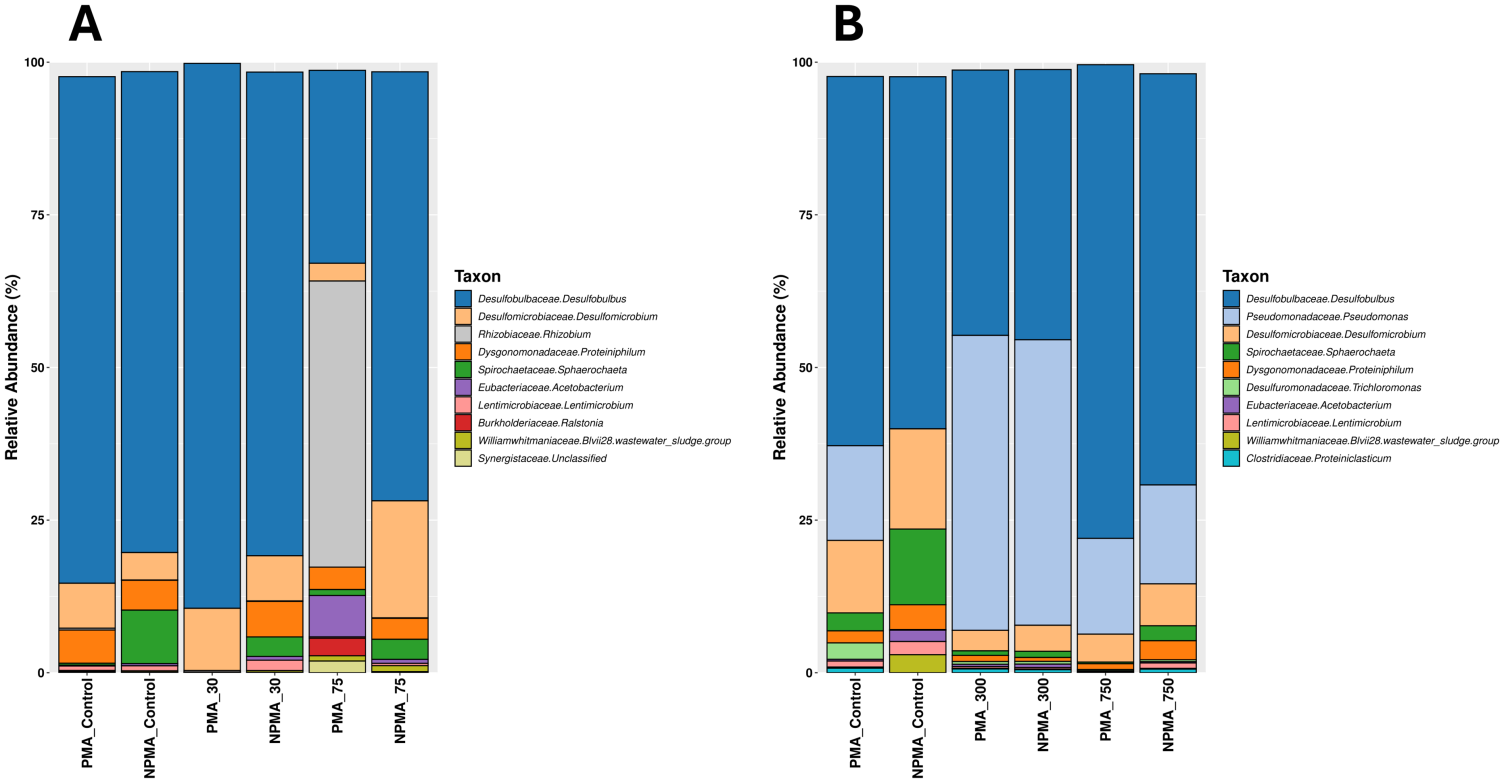
Microbial community compositions of sulfate-reducing biofilms treated with **(A)** lower concentrations of SNP, or **(B)** higher concentrations of SNP. Samples were treated with PMA or remained untreated (NPMA) prior to sample preparation for 16S rRNA gene sequencing. The top 10 most abundant ASVs are shown.

To determine whether similar effects of low SNP doses could also effectively treat existing SRM biofilms, flow cells were established to create active sulfate-reducing biofilms that could be challenged with different concentrations of SNP. The initial flow cell experiment tested concentrations of SNP ranging from 15 to 150 ppm (Figure 3A) alongside an untreated control. Following flow cell inoculation, active biofilms were established in the flow cells within approximately 5 days, after which medium flow was initiated in the system. This action initially lowered sulfide concentrations (due to dilution with medium) but sulfide production increased within the 5 days following the flow regime. It should be noted that prior to biocide dosing, these biofilms that were established in separate flow cells are biological replicates (*n* = 5), all showing similar trends in sulfide production (Figure 3A; Supplementary Figure 1). At day 11, SNP was added at the various concentrations for approximately 10 h, after which sulfide levels dropped in all SNP-treated biofilms; this was not observed in the control (untreated) biofilm (Figure 3A). After the period of SNP treatment, fresh CSBK was then added to each flow cell, and by the following day (approximately 24 h later), sulfide concentrations increased (rebounded) in all treated biofilms, showing that SNP doses ranging from 15 to 150 ppm were only transiently effective. This effect was also observed when these SNP concentrations were tested in separate flow cell experiments (Supplementary Figure 5), confirming the results. Untreated biofilms, and those treated with 30 or 75 ppm SNP were analyzed using 16S rRNA gene sequencing, which revealed that these biofilms were comprised primarily of *Desulfobulbus*, with lower relative abundances of *Desulfomicrobium* and other taxa (Figure 4A), like the community composition of the planktonic culture used as the inoculum for the flow cells (Supplementary Figure 3). PMA-treated samples did not show substantive differences compared to the non-PMA treated samples for the control or the 30 ppm SNP-treated biofilms. For the 75 ppm SNP-treated biofilm samples treated with PMA, *Rhizobium* became enriched in contrast to the non-PMA treated biofilms which was dominated by *Desulfobulbus* like the other samples (Figure 4A).

Since concentrations of SNP up to 150 ppm were only transiently inhibitory to existing SRM biofilms, a second flow cell experiment was carried out wherein established biofilms were untreated, or treated with 300 or 750 ppm SNP to determine whether these higher doses would result in more permanent inhibition of sulfidogenic activity in the biofilms. Like the lower-dose SNP experiment, replicate sulfide-producing biofilms were successfully established (*n* = 3, showing similar trends in sulfide production, Supplementary Figure 1), then treated on day 12 of flow cell operation (Figure 3B) for approximately 10 h with these higher SNP doses. Similar to the lower dose flow cell experiment, sulfide levels initially dropped in the SNP-treated biofilms but not in the untreated biofilm (Figure 3B). Following replenishment with fresh CSBK medium after this treatment time, sulfide concentrations again increased within 1 day, showing that these higher concentrations of SNP were also only transiently effective at inhibiting sulfide production (Figure 3B). Sequencing of the biofilms following the flow experiment showed that *Desulfobulbus* remained as the dominant sulfate-reducer in all samples (PMA-treated or not treated), but in this case, sequences identified as *Pseudomonas* were present as the second most abundant taxon (Figure 4B). There were few differences in the PMA-treated or non-PMA-treated samples from the 300 ppm or 750 ppm SNP-treated biofilms, but the control biofilm (untreated) showed different relative abundances of *Pseudomonas* which was present only in the PMA-treated sample (Figure 4B).

**Figure 5:**
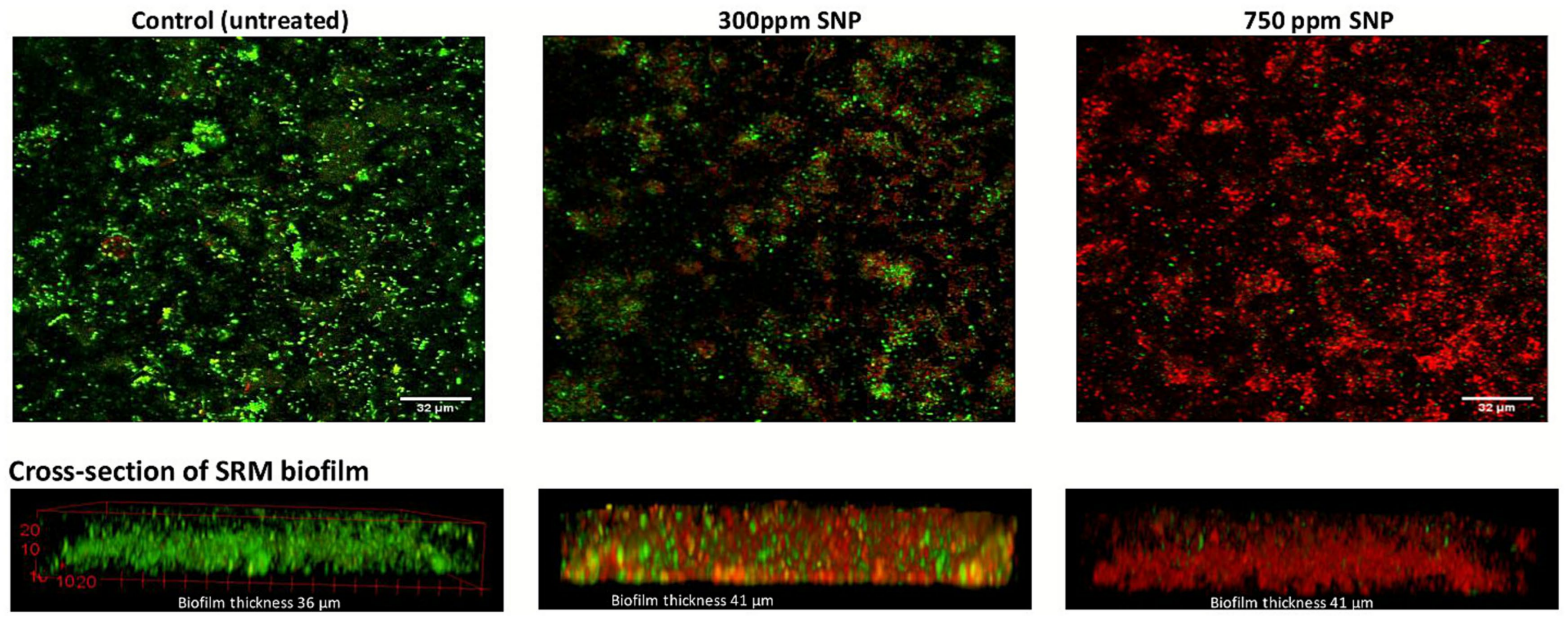
Red and green fluorescence intensities (based on the use of a LIVE/DEAD stain) for SRM biofilms that were untreated or treated with 300 ppm or 750 ppm SNP; top panel shows the top view, while the bottom panel shows the side view of the images obtained using confocal laser scanning microscopy.

Replicate biofilm flow cells from this higher dose SNP experiment were also established to determine the efficacy of biocide penetration into the biofilms, as visualized by confocal laser scanning microscopy. A visual comparison of data sets recorded using identical settings reveals differences in the fluorescence emission from the SYTO 9 (live or viable; membrane-intact) and PI (dead or non-viable; membrane-compromised) channels. Using the LIVE/DEAD stain, an increased green signal indicates more “live” (membrane-intact) cells, while an increased red signal indicates more “dead”, or membrane-compromised cells (Tawakoli et al., 2013). As seen in Figure 5, control (untreated) biofilms show a primarily green signal, suggesting predominantly live (membrane-intact) cells while the 300 ppm-treated biofilm shows a mixture of green and red signal, suggesting the presence of both live and membrane-compromised cells. The 750 ppm-treated biofilm shows mostly membrane-compromised cells, showing effective SNP penetration into the biofilm. However, some green signals in the image suggests that some membrane-intact cells remain in the biofilm even after 750 ppm treatment (Figure 5). An increase in sulfide production soon after the biocide pressure was removed suggests that sulfate-reducing cells can recover after exposure to this higher SNP dose (Figure 3B).

### 3.4 Evaluation of the efficacy of ADBAC on a planktonic SRM culture

ADBAC is well-known to be effective for treating microbial cells in a variety of applications and sectors (Boyce, 2023; Jennings et al., 2015; Merchel Piovesan Pereira and Tagkopoulos, 2019). To verify this efficacy, ADBAC was added to the planktonic SRM culture. Several replicates of this culture were established and demonstrated active sulfide production by 3 days of incubation. On day 3, various concentrations of ADBAC (0, 10, 50, or 100 ppm) were added to the active SRM culture and sulfide was monitored. As seen in Supplementary Figure 6, all tested concentrations of ADBAC inhibited sulfate-reducing activity, as indicated by a continuous decrease in sulfide levels compared to the untreated control. After 20 days of incubation, cultures were then transferred to fresh CSBK medium to determine whether removing the biocide pressure would allow for a resurgence in sulfate-reducing activity. Only the untreated control continued to show sulfide production - this activity was unable to re-establish from all the ADBAC-treated cultures (Supplementary Figure 6). These results show that doses as low as 10 ppm ADBAC were effective at inhibiting sulfide production in the planktonic SRM culture.

### 3.5 Evaluation of the efficacy of ADBAC to treat sulfate-reducing biofilms

**Figure 6:**
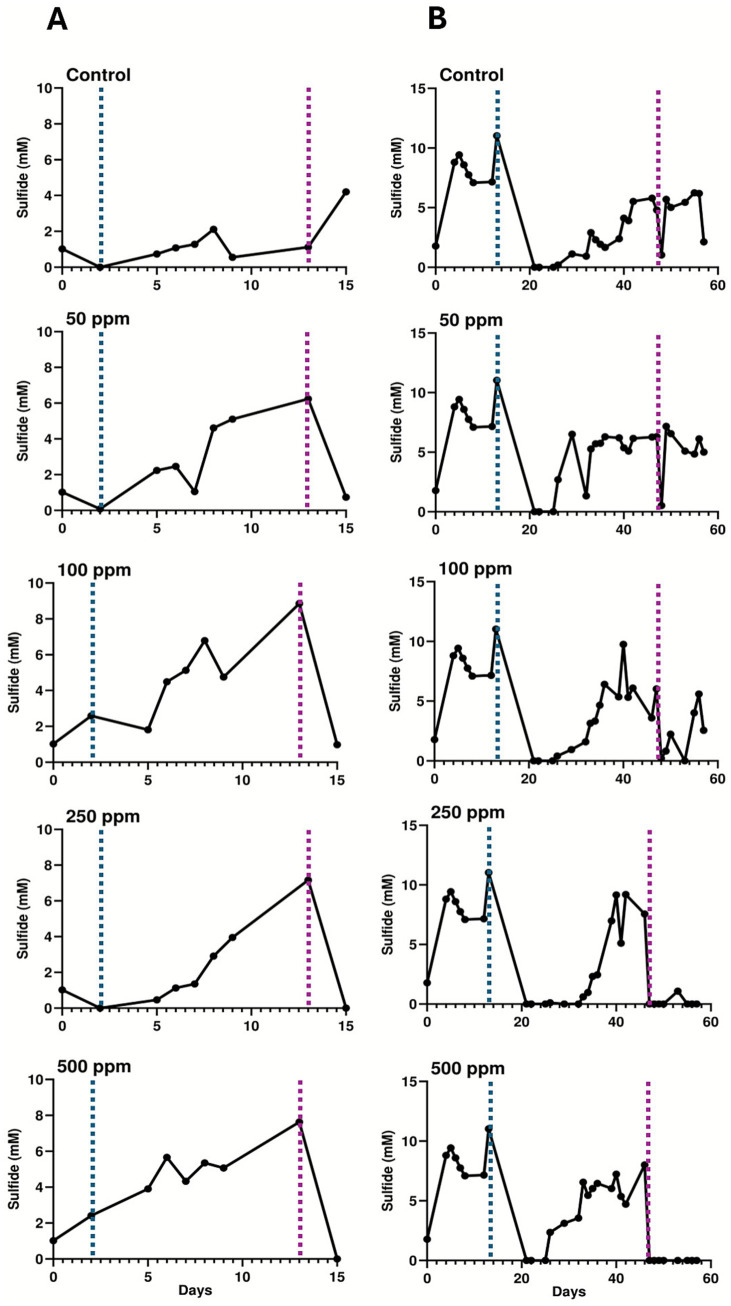
Sulfide production from biofilm flow cells treated with ADBAC at concentrations ranging from 50 to 500 ppm. **(A)** Flow cells were dismantled immediately after the ADBAC addition period. **(B)** Medium was added to flow cells after ADBAC treatment for an additional 10 days before they were dismantled. The first phase of each experiment consisted of biofilm establishment under static conditions after which medium flow through the biofilm was initiated (as indicated by the blue lines). After sulfide production was evident during flow conditions, ADBAC was added to the biofilms (at the point indicated by the red lines) for 14 h. The results of replicate experiments are shown in Supplementary Figures 7, 8.

**Figure 7:**
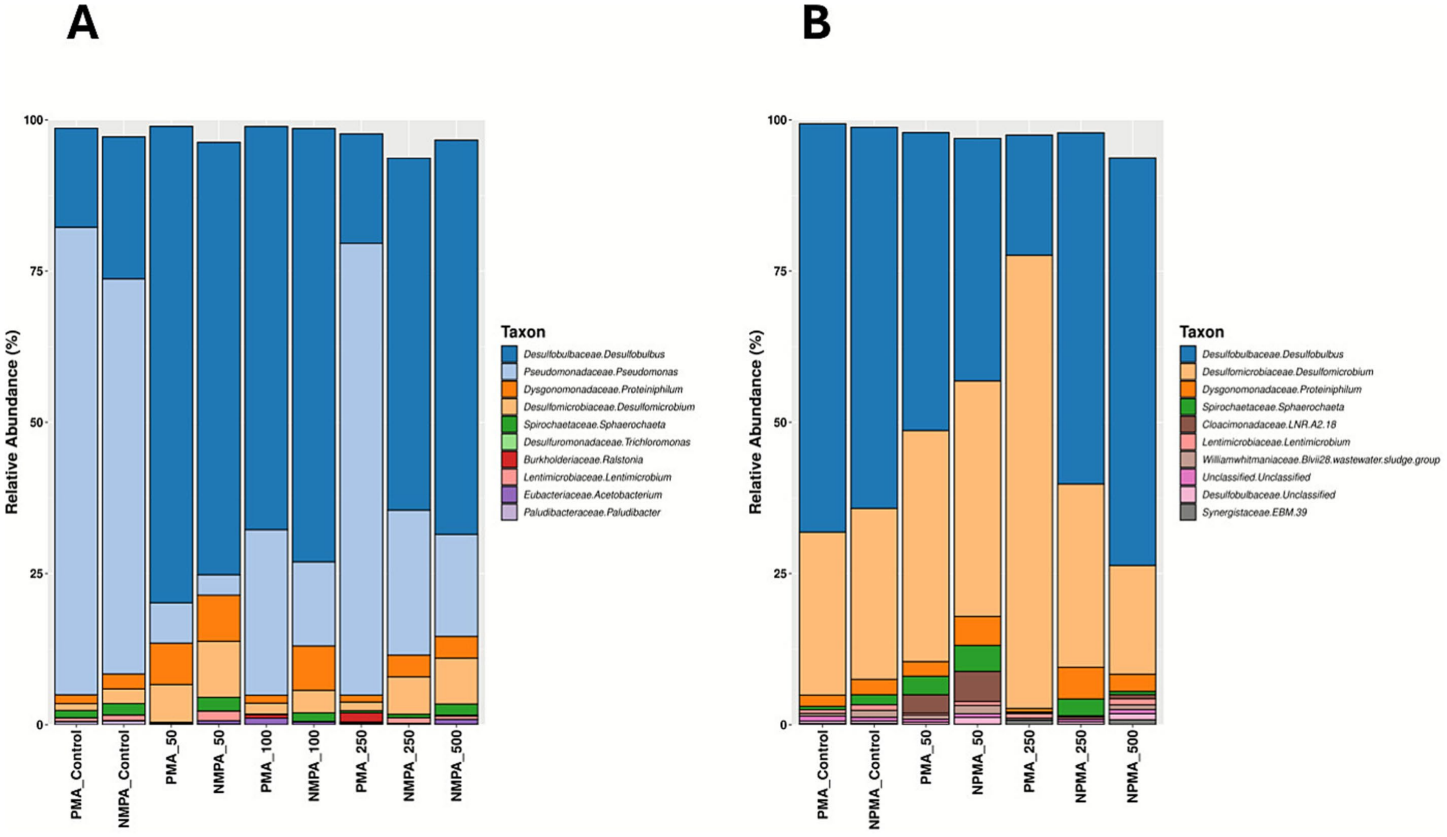
Microbial community composition of sulfate-reducing biofilms treated with various concentrations of ADBAC wherein **(A)** biofilm flow cells were dismantled immediately after ADBAC addition for analysis, or **(B)** biofilms received medium flow for 10 days after biocide treatment before flow cells were dismantled for analysis. Samples were treated with PMA or remained untreated (NPMA) prior to sample preparation for 16S rRNA gene sequencing. The top 10 most abundant ASVs are shown.

Like the SNP tests, biofilms were established in flow cell systems to determine the doses of ADBAC that would inhibit sulfide production from existing sulfate-reducing biofilms. For ADBAC, concentrations of 25 (not shown), 50, 100, 250, and 500 ppm were tested against established biofilms, with different biofilm analyses conducted at the end points of three experiments. Note that for each flow cell experiment conducted, 5 biofilms were established representing biological replicates that were then each treated with a different concentration of ADBAC (Supplementary Figure 1). Further, each experiment was repeated twice to determine reproducibility of the results (Supplementary Figures 7, 8 show the results of repeat experiments). For the first experiment, biofilms were established, treated with varying concentrations of ADBAC, and then the flow cells were opened soon after to determine microbial community composition. In the 5 replicate biofilms, sulfide production reached expected values (e.g., mid-log phase) by day 13 (Figure 6A). At this time, ADBAC was added to the biofilms at various concentrations for 14 h. Sulfide concentrations were measured immediately thereafter, and the flow cells were opened to collect the biofilms for microbial community analysis. In this experiment, sulfide concentrations dropped when all doses of ADBAC were applied compared to the untreated control, where sulfide concentrations continued to increase (Figure 6A). A replicate experiment subsequently conducted showed similar results (Supplementary Figure 7). 16S rRNA gene analysis of the various biofilms showed that *Pseudomonas* were of the highest relative abundance in the control (untreated) biofilms (75–80% relative abundance) with lower relative abundances of *Desulfobulbus* and other taxa (Figure 7A). However, when ADBAC was added at 50 or 100 ppm doses, *Desulfobulbus* became dominant (with or without PMA treatment). When 250 ppm ADBAC was added to the biofilms, the PMA-treated sample showed an increase in relative abundance of *Pseudomonas* compared to *Desulfobulbus*, while the opposite was true when PMA was not added (Figure 7A). DNA could not be extracted from the biofilm samples treated with 500 ppm ADBAC and then treated with PMA suggesting that only membrane-compromised/dead cells were present.

For the second flow-cell experiment testing ADBAC, the aim was to assess whether biofilms treated with ADBAC could resume sulfide production after ADBAC was removed from the system. An initial flow cell experiment was conducted to assess the reproducibility of sulfide production from all 5 biofilms, and the resumption of sulfide production following ADBAC treatment. Following ADBAC treatment, the biofilm treated with 50 ppm ADBAC resumed sulfide production, while all other concentrations inhibited further sulfide production (Supplementary Figure 8). As microbial community analysis was not carried out for this initial experiment, a second similar experiment was carried out that also assessed microbial community composition (Figures 6, 7). Like the first experiment, sulfide production, though variable across all biofilms, continuously increased following the introduction of CSBK medium (Figure 6B; Supplementary Figure 1). On day 46, each biofilm was then treated for 14 h with ADBAC at different doses. The temporary decrease in sulfide production observed on the day of ADBAC treatment was likely due to its dilution caused by adding medium, but sulfide production levels increased after the introduction of medium. Biocide addition resulted in a decrease of sulfide concentrations when ADBAC was added, similar to the previous experiment (Figure 6A, Supplementary Figure 7). To observe whether sulfate-reducing activity of treated biofilms could re-establish after treatment, CSBK medium was re-introduced for 10 days. In the 50 ppm ADBAC-treated biofilm, sulfide rebounded quickly, in a similar manner to the untreated control, showing that this concentration was ineffective at permanently inhibiting sulfide production from this biofilm, also previously seen (Supplementary Figure 8). The 100 ppm ADBAC treatment also showed a sulfide rebound, but in a slightly delayed manner (Figure 6B); this result differed from the previous experiment that showed no sulfide production following 100 ppm treatment (Supplementary Figure 8). In contrast, both the 250 ppm and 500 ppm biocide treated biofilms did not show sulfide production within 10 days after medium re-introduction, showing that these concentrations of ADBAC were comparatively effective at inhibiting sulfate-reducing activity and confirming the results of the replicate flow cell experiment (Supplementary Figure 8). After 10 days of medium flow, the flow cells were opened, and the biofilms were sampled for microbial community analysis by 16S rRNA gene sequencing. In this biofilm experiment, *Desulfobulbus* was the most abundant taxon, followed by *Desulfomicrobium*, as seen in the untreated biofilms (Figure 7B). When 50 ppm ADBAC were added, the community showed a decrease in the relative abundance in *Desulfobulbus* and an increase in *Desulfomicrobium*, which was even more pronounced when 250 ppm ADBAC were added (in the PMA-treated samples). Like the previous experiment, DNA could not be extracted from the PMA-treated biofilm sample retrieved from the 500 ppm ADBAC treatment (Figure 7B).

**Figure 8:**
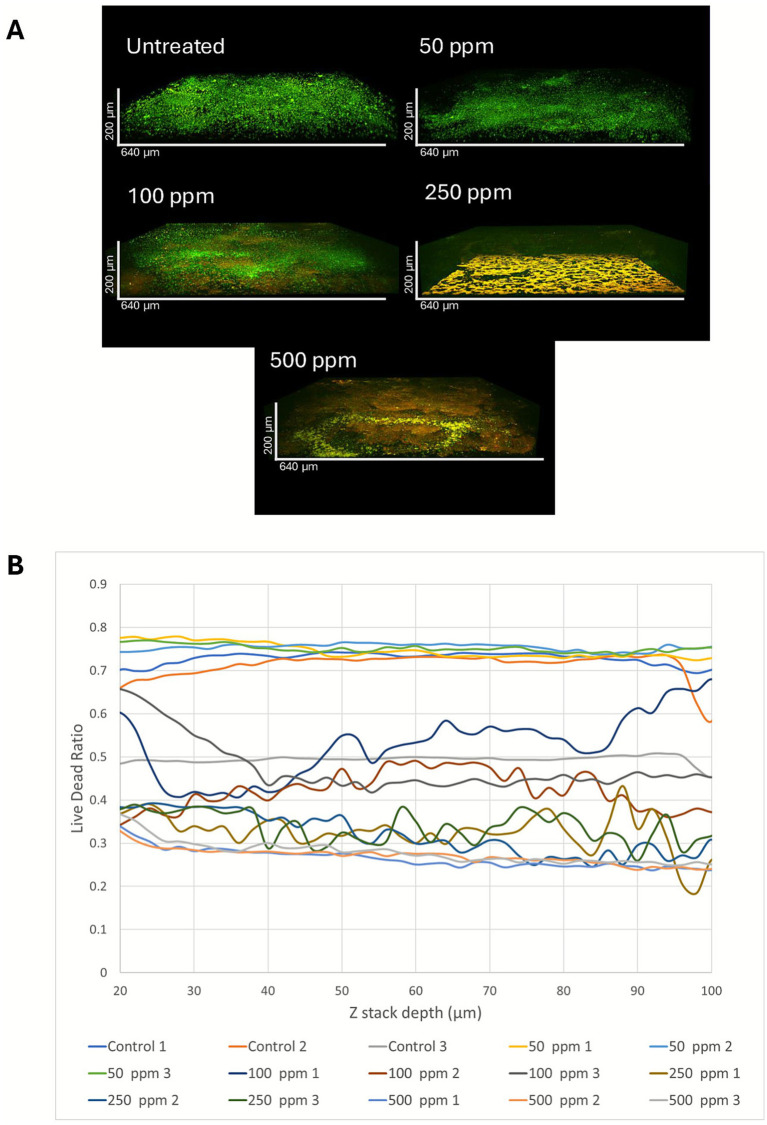
**(A)** Red and green fluorescence intensities (based on the use of a LIVE/DEAD stain) shown in 3D projection for biofilms treated with various concentrations of ADBAC, as analyzed by two-photon microscopy. **(B)** Ratio of the integrated intensity in the SYTO 9 (viable) channel divided by the sum of the integrated intensity of the SYTO 9 (viable) and PI (non-viable) channels across multiple *z*-stacks, as determined using CellProfiler. Ratio values closer to 1 on the *y*-axis indicate a higher proportion of viable cells, while those closer to 0 indicate a higher proportion of non-viable cells.

A final flow cell experiment was established in an identical manner to obtain samples for microscopy analysis (Supplementary Figure 7). Supplementary Figure 1 shows the results of the sulfide production from the 5 replicate biofilms prior to ADBAC addition, again showing a general upward trend of sulfide production despite variability amongst each biological replicate. Following the treatment of each flow cell with different concentrations of ADBAC, medium was allowed to flow through each biofilm for 30 min then biofilms in the flow cells were stained with the LIVE/DEAD Biofilm Viability kit for analysis by two-photon microscopy. Figure 8 illustrates representative 3D visualizations of the imaging data, with green representing SYTO 9 (live) and magenta representing PI (green and magenta were chosen as they are more visually accessible for people with color vision deficiencies). Cross-sectional views (edge on view of the Z-stack) are shown, allowing qualitative inspection of the profile of the various biofilms.

The LIVE/DEAD kit should reflect the relative abundance of viable cells in the total population. We noted that the overall SYTO 9 fluorescence decreased when the samples were treated with biocide. Figure 8A provides representative 3D visualizations for comparison across conditions. Each channel is displayed using identical settings for brightness and contrast. The untreated and 50 ppm ADBAC-treated biofilms emit primarily in the SYTO 9 channel indicating live cells (shown as green) and corresponding closely to the flow cell observations (Figure 6). ADBAC added at 100 ppm yielded signal primarily in the SYTO 9 channel as well, but also showed discrete regions where fluorescence was higher in the PI channel (non-viable, shown as magenta), aligning with the sulfide measurements showing more of a delay in the resurgence of sulfate-reducing activity. Finally, the 250 and 500 ppm treatments showed little emission in the SYTO 9 channel (live, detected as green), aligning with the flow cell data showing no sulfide activity rebounding following treatment with these ADBAC concentrations.

To compare the relative SYTO 9 and PI fluorescence emission for each condition, the ratio of the integrated intensity in the SYTO 9 channel was then divided by the sum of the integrated intensity of the SYTO 9 and PI channels. This normalization was required because the overall intensity in both the STYO 9 and PI channels decreased in the treated samples. The resulting ratio varied between 0 and 1, with 1 being the case when all the intensity is derived from the SYTO 9 channel only and conversely, when all the signal is derived from PI signal only.

Figure 8B illustrates a plot of the ratio of the integrated intensity (0–1) by z-slice (z-stack 20 to 100, with 20 being closer to the coverslip). A plot of the ratios for each conditions demonstrates a systematic trend, with a decreasing ratio when ADBAC was applied. A decreasing ratio implies that the SYTO 9 fluorescence emission is decreasing relative to PI emission, as would be expected when the cells become less viable. As shown in Figure 8B, the control and 50 ppm as well as the 250 ppm and 500 ppm results track closely, while the 100 ppm is intermediate between these two extremes. Thus, both the qualitative and quantitative imaging results from the ADBAC biofilm experiments align closely with the results of the biological assays.

## 4 Discussion

Biofilms can readily form in aquatic systems, some of which can be detrimental to industrial infrastructure, causing fouling or corrosion that can inhibit functionality and/or structural integrity (Ma et al., 2020; Rubio et al., 2015; Tuck et al., 2022; Vigneron et al., 2016). In many sectors, biocides are used to inhibit or kill detrimental microorganisms, but biocide dose testing often uses water samples or planktonic cultures rather than surface-attached communities. Research on biofilms using clinically relevant bacteria have convincingly shown that organisms living in biofilms have significantly decreased susceptibility to antimicrobials (Cao et al., 2015; Ciofu et al., 2022; Grobe et al., 2002; Ioannou et al., 2007). This effect occurs for many reasons, including the presence of a highly protective ECM that can sorb or neutralize external substances, and the fact that biofilms are usually comprised of various sub-populations that are dormant or grow very slowly thus are not affected by antimicrobials that target growing cells (Ciofu et al., 2022; Liu et al., 2024). Inadequately treating detrimental biofilms (such as with ineffective dosing) can lead to the development of tolerance or antimicrobial resistance (Baig et al., 2023; Boyce, 2023; Charron et al., 2023; Ciofu et al., 2022; Coombs et al., 2023).

Presumably, similar concepts apply to biofilms outside of the clinical environment, but less research overall has been done with organisms that are associated with many industrial settings where biofilm growth can be a problem. However, a handful of studies have used Robbins devices or CDC bioreactors to assess biofilm growth and test biocide concentrations, particularly related to preventing microbial corrosion in the oil and gas sector (Broussard et al., 2017; Keasler et al., 2009; Salgar-Chaparro et al., 2020; Jones et al., 2025). Other studies used SRM isolates, such as *Desulfovibrio vulgaris*, or enriched anaerobic consortia from oilfields that grown on carbon steel coupons in the laboratory to determine the effects of biocides (and biocide enhancers in some cases) for preventing corrosive biofilms from forming under non-flow conditions (Jia et al., 2017; Li et al., 2016; Xu et al., 2022). In this study, we sought to investigate the effects of biocides on existing biofilms of sulfate-reducing microorganisms. To our knowledge, the use of a flow cell system like that used here to establish sulfate-reducing biofilms that can then be assessed for biocide efficacy has not been previously reported.

Sulfate-reducing microorganisms, that produce sulfide as their respiratory end product, can grow in many aquatic ecosystems associated with man-made infrastructure such as marine vessels, mooring structures, oil production platforms, wind energy monopiles, and sewer and other wastewater systems (Ma et al., 2020; Rubio et al., 2015; Sun et al., 2014; Tuck et al., 2022; Vigneron et al., 2016; Wade et al., 2011; Zhang et al., 2022). In these types of environments, SRM metabolism can lead to negative operational issues such as souring and materials corrosion thus are an important group of microorganisms to target for biofilm control. To initiate the study described herein, we enriched an anaerobic microbial community from pipeline pigging sludge under sulfate-reducing conditions. We used propionate as the carbon source and electron donor to develop an enrichment culture that grew relatively quickly and could be easily maintained over the time frame of the experiments. The culture contained comparatively high relative abundances of *Desulfobulbus*, a known propionate-utilizing SRM (El Houari et al., 2017) but was also comprised of other sulfate-reducers such as *Desulfomicrobium*, facultative anaerobes, and fermentative bacteria (Supplementary Figure 3). Despite having non-sulfate-reducing taxa present, the culture showed reliable sulfate-reducing activity. It should be noted that other sulfidogenic samples, mixed cultures, or isolates may respond differently to the biocide results described here as it is well known that different sources, species, and chemical environments (including nutrient levels), will impact responses to biocide treatments (Salgar-Chaparro et al., 2020; Tuck et al., 2022; Welikala et al., 2024).

A flow cell system was used to establish SRM biofilms from this enrichment culture that were then treated with different doses of either SNP, a proposed new “green” biocide, or ADBAC, an industry-accepted biocide that is widely used across many sectors. Under the conditions established in this study, we found that SNP was only transiently effective in mitigating sulfide production from established SRM biofilms up to doses of 750 ppm (Figure 3). LIVE/DEAD cell staining and confocal microscopy analysis showed that SNP was able to penetrate biofilms (based on a primarily red signal indicating membrane-compromised cells when biofilms were treated with 750 ppm SNP, Figure 5), however removal of biocide from the flow cell system resulted in a rapid resurgence of sulfide production (Figure 3). In contrast, 15 ppm SNP effectively inhibited sulfate-reducing activity in planktonic incubations of the same SRM culture, confirming a previous study showing that similar low SNP doses were effective against planktonic organisms (Fida et al., 2018). Thus, in this present study, SNP appeared to have a bacteriostatic rather than a bacteriocidal effect on biofilms, with the biofilm-associated cells tolerating ~50 times higher SNP concentrations than the planktonic cells. Because SNP can also chemically react with sulfide, this may have occurred once SNP was added to the biofilms, leaving less “free” SNP available to interact with biomolecules. Previous studies showed that low concentrations of SNP can work synergistically with nitrate to inhibit souring in sand-packed columns (Fida et al., 2018) or biofilm sewer bioreactors (Zhang et al., 2022), thus this proposed “green” biocide may find use as a treatment enhancer (Jenneman and De Leόn, 2022) rather than as a standalone treatment chemical for preventing souring or corrosion due to SRM activity.

The established SRM biofilms that were treated with ADBAC showed more varied responses to the different doses tested, which was particularly evident when biocide challenges were removed and replaced with fresh medium. ADBAC added at 50 ppm did not substantially affect the existing SRM biofilms, as both sulfide responses and microscopic images were very similar to the untreated control (Figures 6, 8). ADBAC added at 100 ppm to existing biofilms showed some delay in the resumption of sulfide production compared to the untreated control (Figure 6B), with two-photon microscopic analysis showing some evidence of membrane-compromised cells (appearance of magenta within the green signal; Figure 8A). However, mixed results were obtained with 100 ppm ADBAC treatments (Figure 6B, Supplementary Figure 8) thus this concentration cannot be deemed reliably effective against SRM biofilms. The 250 ppm and 500 ppm ADBAC treatments of the SRM biofilms were effective in that sulfide did not resume following removal of the biocide pressure over a 10-day period (Figure 6B, Supplementary Figure 8), also confirmed by two-photon microscopy showing a substantial decrease in the green (live cell) signal (Figure 8). Overall, the quantitative two-photon microscopy analysis also confirmed the biochemical data obtained with ADBAC-treated biofilms.

Although confocal microscopy is widely applied for biofilm imaging, two-photon (multiphoton) imaging is relatively under-used. Compared to confocal microscopy, two-photon microscopy is less affected by absorption and scattering and offers superior optical sectioning, and properly optimized, is less damaging to the sample. It also is more efficient, as the fluorescence emission can be measured closer to the objective compared to a confocal system, where there are more losses due to descanning and the need for spatial filtering with a confocal pinhole. Last, the detectors often have larger surface areas, also allowing capture of scattered photons that would be rejected by a confocal microscope. Thus, two-photon imaging can be enormously helpful in imaging biofilms to confirm biochemical measurements.

After biocide treatments of the SRM biofilms, they were sampled and prepared for 16S rRNA gene sequencing to identify biofilm-associated microorganisms in the absence or presence of biocide. Each biofilm sample was treated with PMA which is known to bind to DNA in membrane-compromised cells or that is present outside of cells, allowing for DNA extraction only from viable (membrane-intact) cells (Nocker et al., 2007; Wang et al., 2021). Theoretically, taxa identified in PMA-treated samples are thus a reflection of the viable cells in a community. Each biofilm sample was also subject to DNA extraction without PMA treatment, which should allow for the identification of both viable and non-viable taxa. Wang et al. (2021) recently conducted a study comparing the use of PMA to distinguish between viable and non-viable cells and found that this approach was reliable for pure cultures or simple, synthetic communities, but yielded inconsistent results for more complex communities sampled from various ecosystems. With that caveat in mind, along with all known biases of primer-based amplicon sequencing (Schirmer et al., 2015), we compared the microbial taxa identified in PMA vs. non-PMA (NPMA) treated biofilm samples to help determine whether any taxa within the biofilms were particularly susceptible to biocide treatment.

For biofilms treated with SNP, wherein all treatments up to 750 ppm displayed a resurgence of sulfide production after biocide removal, PMA- and non-PMA treated samples did not show many differences in the relative proportions of taxa, which showed *Desulfobulbus* as the dominant taxon in almost all biofilms (with the exception of *Rhizobium* appearing dominant in the 75 ppm + PMA treatment; an anomaly that we cannot explain) (Figure 4A). For the lower dose SNP experiment, *Desulfomicrobium* generally remained as the second most abundant taxon. For the biofilm experiment performed with higher concentrations of SNP (300 and 750 ppm), *Desulfobulbus* was also dominant in most cases, but the culture shifted to showing *Pseudomonas* as the second most abundant taxon (rather than *Desulfomicrobium* as was seen in the lower dose SNP experiments) (Figure 4). *Pseudomonas* was not abundant in the planktonic culture used for inoculations (<0.2% relative abundance), so it is not clear why this organism became more abundant, particularly in the 300 ppm SNP treatments. Some *Pseudomonas* spp. can use NO as a signaling molecule for biofilm dispersal (and thus possess resistance mechanisms; Chautrand et al., 2022) so it is possible that these organisms have a better ability to withstand NO released by SNP while the biofilm was exposed to this chemical. However, sulfate-reducing activity in the biofilms was not impeded by the SNP doses tested, so SRM like *Desulfobulbus* remained active in the biofilm as well. Unexpectedly, *Pseudomonas* also appeared as a dominant taxon in the first biofilm experiment performed to test the effects of ADBAC (Figure 7A), as evident in the control (untreated) biofilms, so it is possible that this taxon was present in high relative abundance in the planktonic culture used for the experiments. Unfortunately, the planktonic culture used as the flow cell inoculum was not sequenced when these two experiments were performed so we cannot clearly explain why *Pseudomonas* became prevalent in two of the four biofilm experiments conducted. In any case, when ADBAC was added to the biofilms at 50 ppm and 100 ppm doses, *Desulfobulbus* appeared as the most dominant taxon when biofilms were sampled immediately after biocide treatment (Figure 7A). In the PMA-treated biofilms, theoretically indicative of the viable population, slightly higher relative proportions of *Pseudomonas* were present than in the non-PMA-treated samples. Notably, biofilms collected from the 250 ppm ADBAC treatment then treated with PMA prior to DNA extraction showed a substantial increase in the relative abundance of *Pseudomonas* compared to the non-PMA-treated samples. In the subsequent flow cell experiment testing the effects of sulfate-reducing activity recovery following ADBAC treatment, biofilms were sampled 10 days following the cessation of biocide treatment and amendment with medium flow only. This flow cell experiment again showed *Desulfobulbus* as highly abundant in the biofilm but in this experiment *Desulfomicrobium* was the second most abundant taxon in the biofilms (Figure 7B). The relative proportions of the 50 ppm ADBAC-treated biofilms were similar to the untreated control (PMA-treated or not), aligning with the observation of sulfide production resurging after fresh medium was added (Figure 6B). PMA-treated biofilms retrieved from the 250 ppm ADBAC treatment differed substantially from the non-PMA-treated sample wherein *Desulfomicrobium* was present in high relative abundance compared to *Desulfobulbus*. This decrease in the relative abundance of *Desulfobulbus* when 250 ppm ADBAC-treated biofilms were sequenced (seen with both flow cells experiments testing ADBAC), coupled with a cessation of sulfate-reducing activity under these conditions suggests that *Desulfobulbus* played an important role in reducing sulfate in the biofilms and that this SRM was highly susceptible to ADBAC treatment. Even though *Desulfomicrobium* increased substantially in its relative abundance in the 250 ppm ADBAC treated biofilms, sulfide production did not resume (Figures 6B, 7B). In this case, inhibiting *Desulfobulbus* as the propionate-using sulfate-reducer in the biofilms likely reduced the pool of acetate in the system (not measured), potentially also inhibiting sulfate-reducing acetate-users such as *Desulfomicrobium*. This observation points to the fact that it is critical to make empirical measurements of microbial activity rather than rely solely on microbial community sequencing data to make conclusions about how a system might behave. Further, any observed changes in relative abundances observed during these experiments could be due many other factors associated with sample processing and amplicon sequencing such as DNA extraction biases, chemical contaminants interfering with PCR reactions, primer biases, and sequencing errors (Schirmer et al., 2015). Overall, the use of PMA to help identify viable cells in the biofilms following biocide treatment gave mixed results, similar to that reported by Wang et al. (2021). A study examining anaerobic biofilms in sewers observed the stratification of sulfate-reducers and methanogens in the biofilms (Sun et al., 2014). The sulfate-reducers *Desulfobulbus*, *Desulfomicrobium*, and *Desulfovibrio* were primarily associated with the biofilm surface where most sulfate-reducing activity was measured, while methanogens and methane production were deeper in the biofilm (Sun et al., 2014). Future studies examining the stratification of taxa in the biofilms established here could help further explain the tolerances and susceptibilities of the dominant sulfate-reducers *Desulfobulbus* and *Desulfomicrobium* to biocide treatments (Mohanakrishnan et al., 2011).

Overall, the results revealed that higher doses of biocides are needed to effectively treat existing SRM biofilms compared to planktonic cells, confirming many previous studies. Further, using a flow cell system, we found that adding biocides at some concentrations are only transiently effective, as activity can readily rebound once biocides are removed from a flowing system. In many industrial applications, biocides are added at defined intervals and if underdosing occurs, this can lead to wasted costs and persistent problems. Notably, underdosing can also lead to increased tolerance of microorganisms to antimicrobials including biocides like QACs, leading to an overall decrease of its effectiveness and an increase in antimicrobial resistance (Baig et al., 2023; Boyce, 2023; Charron et al., 2023; Ciofu et al., 2022; Xu et al., 2022). It is thus critical to study the effects of chemical treatments on surface-attached microbial communities rather than planktonic communities in aquatic environments to ensure the proper mitigation of problematic biofilms and minimize further environmental implications such as the development of antimicrobial resistance.

## Data Availability

Amplicon sequencing data is available in GenBank (NCBI) under BioProject accession number PRJNA1284394.

## Footnotes

1
https://github.com/kiragoff
