# Supplementary material for: Determining biocide efficacy for treating established sulfate-reducing biofilms using flow cell systems
Source: Front Microbiol. 2026 Jan 23;16:1646177. doi: 10.3389/fmicb.2025.1646177 (PMC12876176; doi:10.3389/fmicb.2025.1646177)
Supplement: Supplementary file 1 [file Data_Sheet_1.docx]

**Determining biocide efficacy for treating established sulfate-reducing biofilms using**

**flow cell systems**

Gloria N. Okpala^1#^^, Anna L. Walker^1#^, Craig Brideau^2^, Pina Colarusso^2^, and Lisa M. Gieg^1^

^1^ Department of Biological Sciences, University of Calgary, Calgary, AB, Canada

^2^ Live Cell Imaging Laboratory, Department of Physiology & Pharmacology, Cumming School of Medicine, University of Calgary, Calgary, AB, Canada

# These authors contributed equally to this work and share first authorship.

^Current address, Baker Hughes Canada, Calgary, AB

*Corresponding author, lmgieg@ucalgary.ca

**1 Supplementary Table**

**8 Supplementary Figures**

**Supplementary Table 1.** Summary of the number of raw reads and quality-controlled reads* (used for creating relative abundance figures) for 16S amplicon sequencing results (Figs. 4 and 7).

| **Figure** | **# raw reads** | **# quality-controlled reads** |
| --- | --- | --- |
| **Figure 4a (SNP)** |  |  |
| PMA_Cont | 80736 | 58467 |
| NPMA_Cont | 98590 | 76347 |
| PMA_30 | 87874 | 67466 |
| NPMA_30 | 96856 | 70302 |
| PMA_75 | 95071 | 66638 |
| NPMA_75 | 93335 | 62494 |
| **Figure 4b (SNP)** |  |  |
| PMA_Cont | 127168 | 83918 |
| NPMA_Cont | 129603 | 82938 |
| PMA_300 | 56419 | 41354 |
| NPMA_300 | 97715 | 74274 |
| PMA_750 | 121578 | 90290 |
| NPMA_750 | 119598 | 84498 |
| **Figure 7a (ADBAC)** |  |  |
| PMA_Cont | 242250 | 113428 |
| NPMA_Cont | 200440 | 107241 |
| PMA_50 | 193679 | 100815 |
| NPMA_50 | 250362 | 126472 |
| PMA_100 | 177781 | 119931 |
| NPMA_100 | 251244 | 119907 |
| PMA_250 | 131352 | 67608 |
| NPMA_250 | 247277 | 96635 |
| NPMA_500 | 292302 | 139969 |
| **Figure 7b (ADBAC)** |  |  |
| PMA_Cont | 135408 | 61242 |
| NPMA_Cont | 138794 | 73266 |
| PMA_50 | 121824 | 71869 |
| NPMA_50 | 134297 | 62971 |
| PMA_250 | 140170 | 101700 |
| NPMA_250 | 138860 | 75040 |
| NPMA_500 | 125281 | 62265 |

*Raw reads were processed using a filter and trim function that considered sequencing errors based on Q scores (<2 errors), a minimum length filter (<75 reads), and removal of 'n' bases. Forward and reverse reads were then merged, chimeras were removed, and sequences outside the expected range (350-400 bp) were removed, yielding the quality-controlled number of reads (for R code pipeline, see https://github.com/kiragoff). PMA = samples treated with propidium monoazide; NPMA = untreated samples.


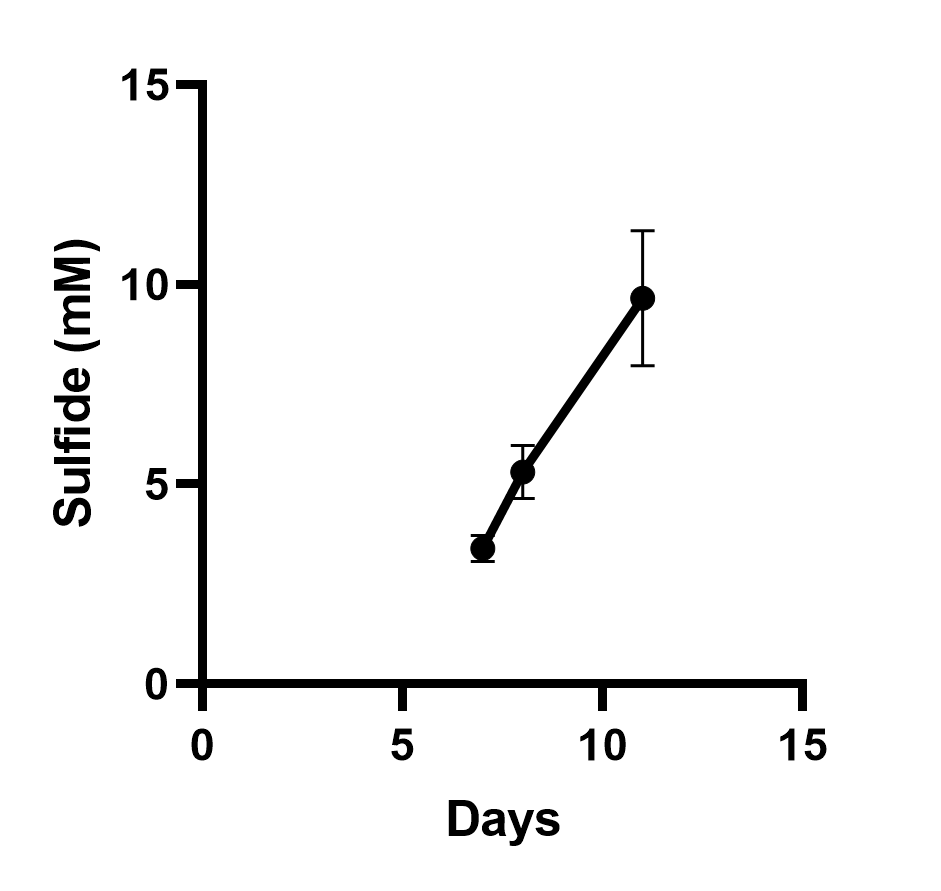

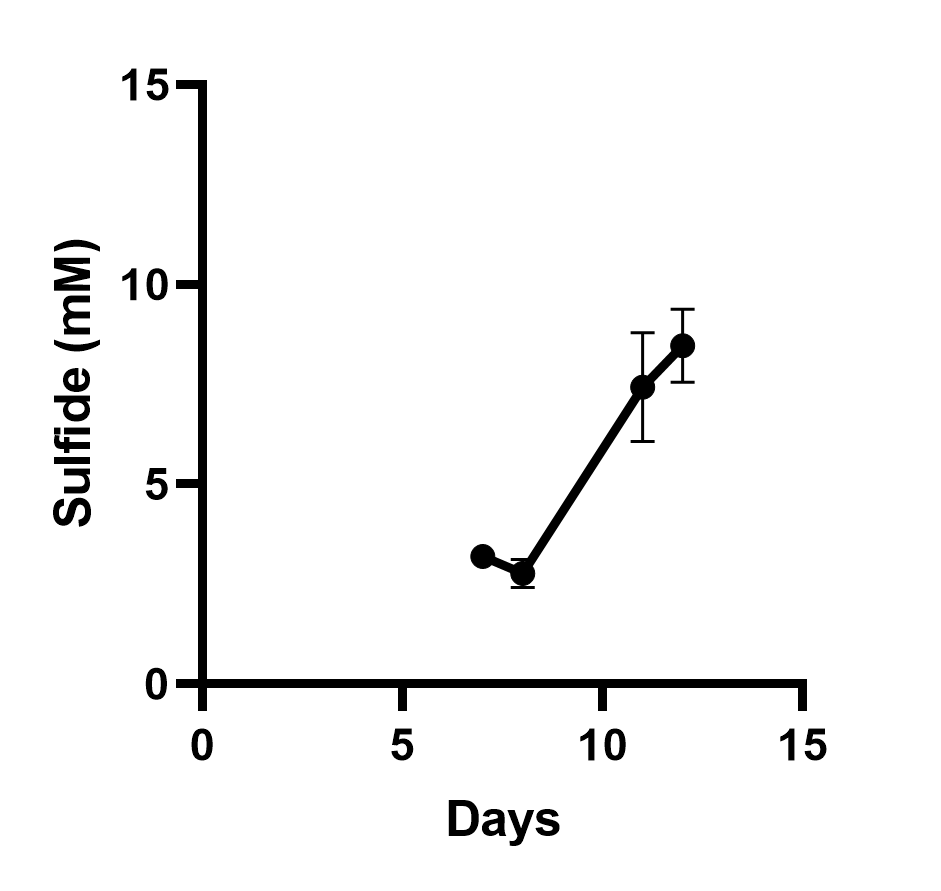


**A**

**B**


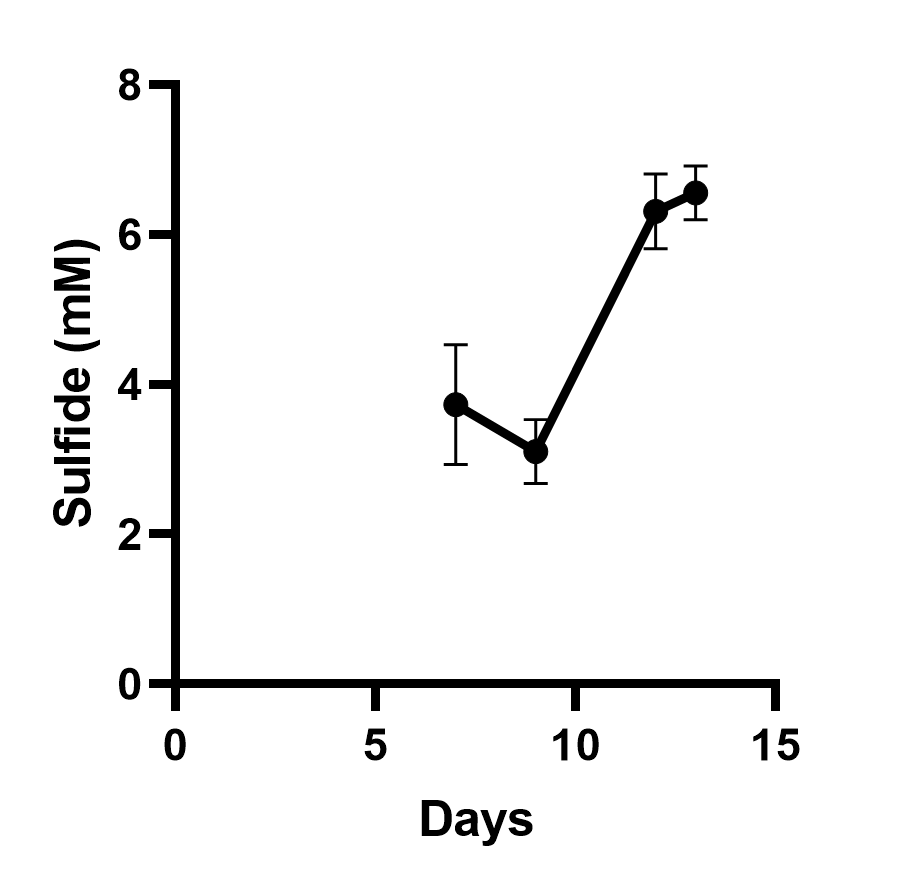


**C**


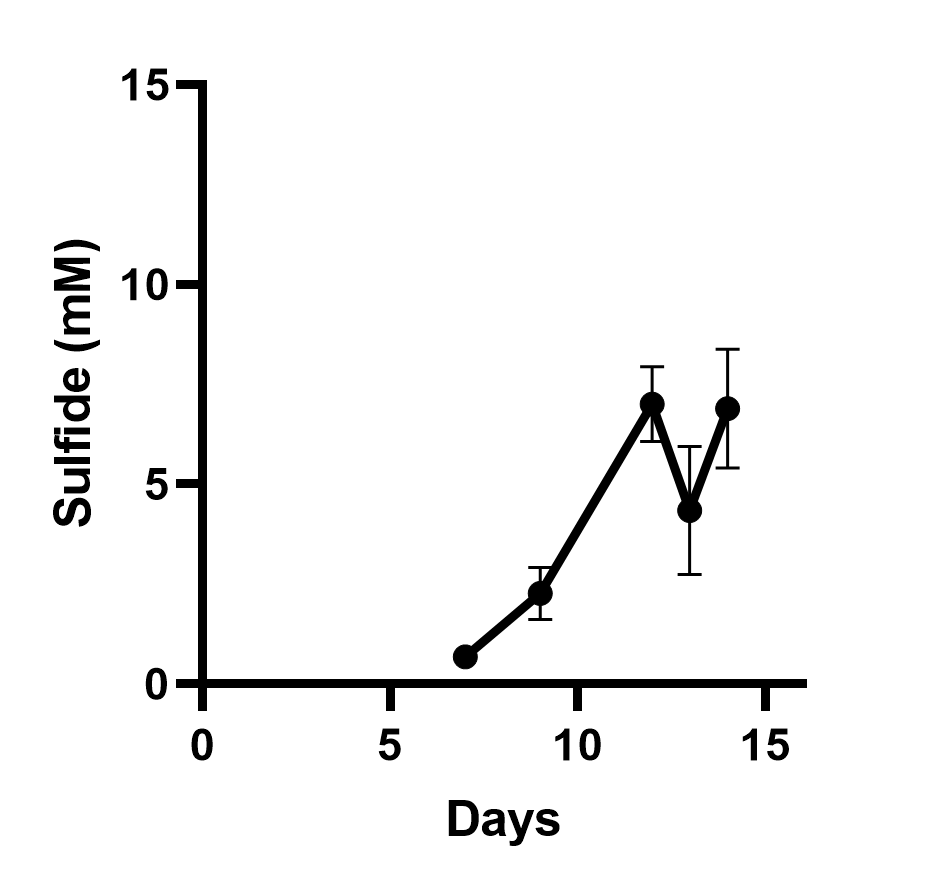


**D**


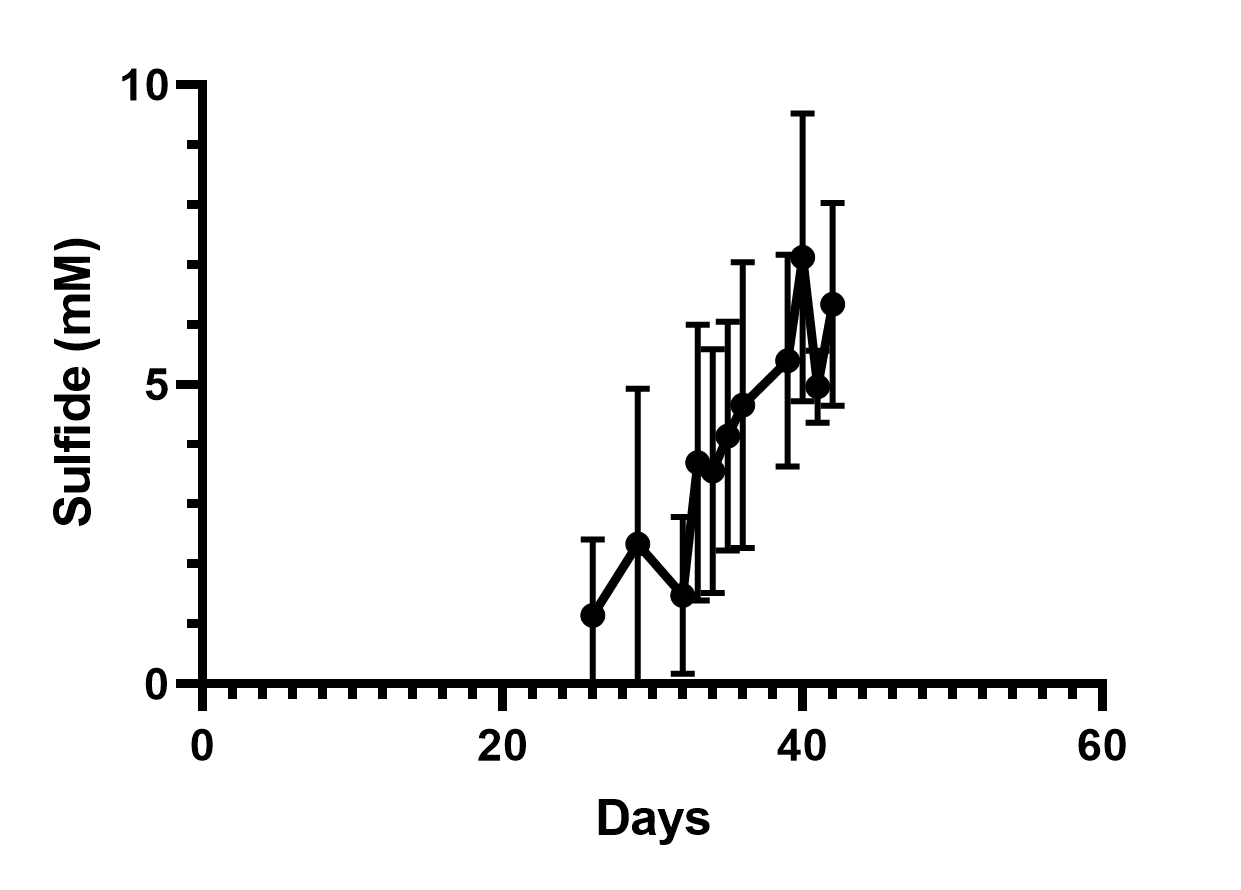

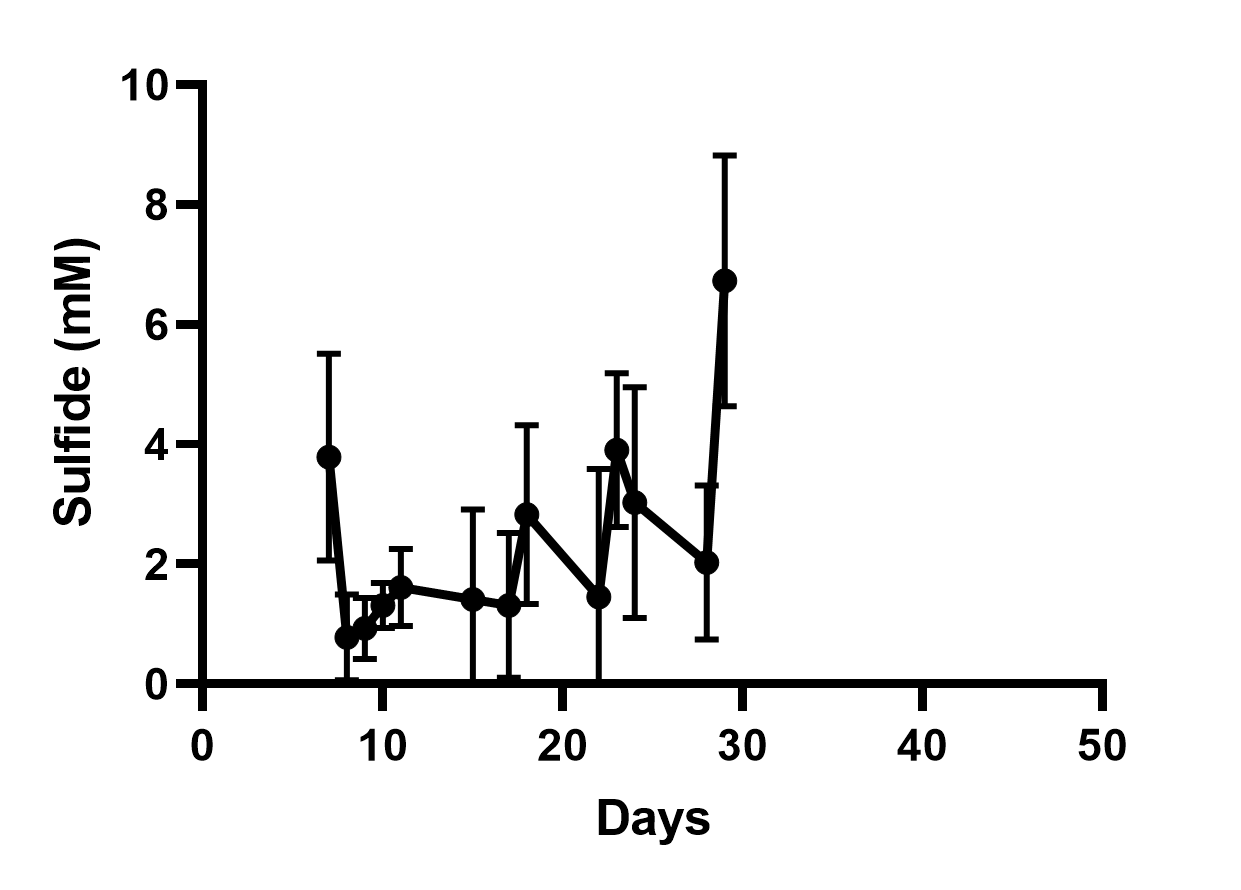

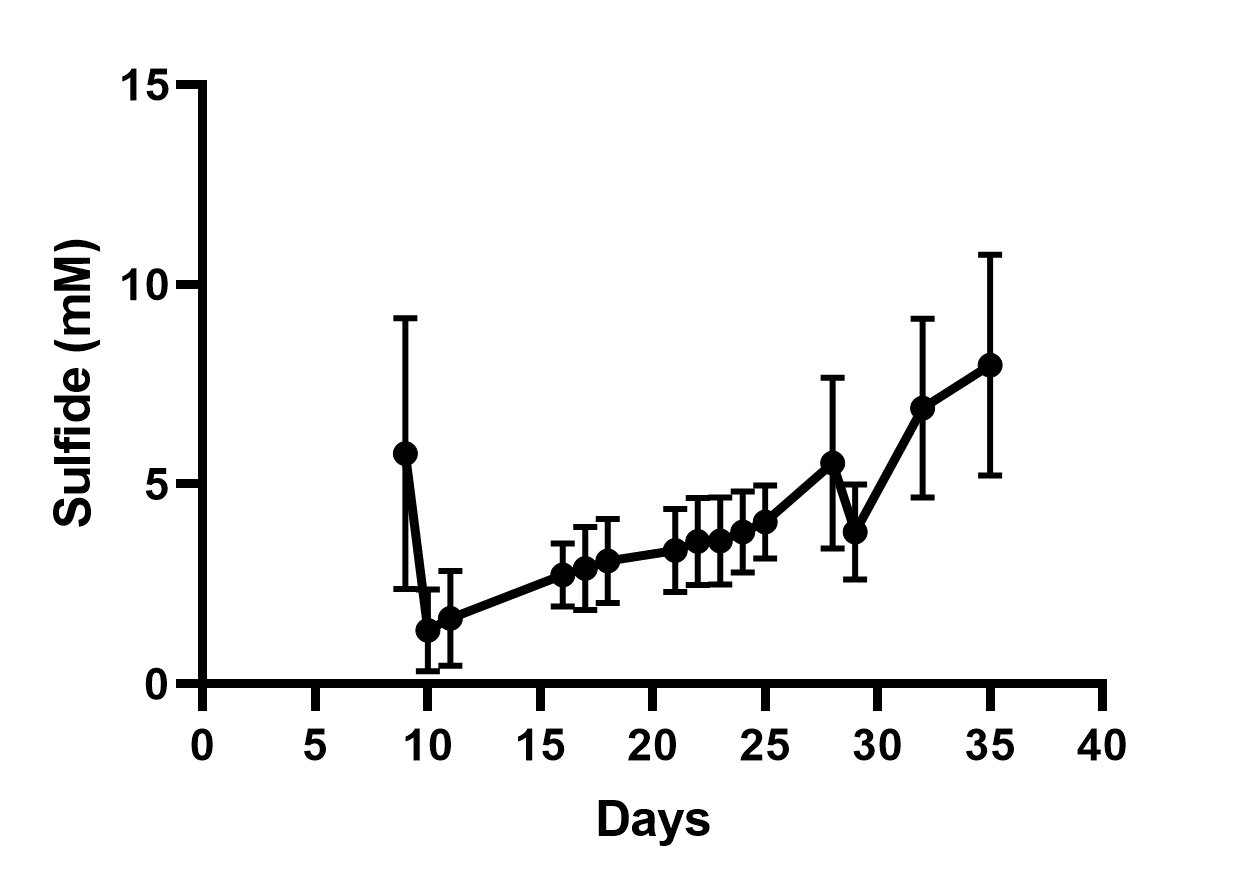


**E**

**F**

**G**

**H**


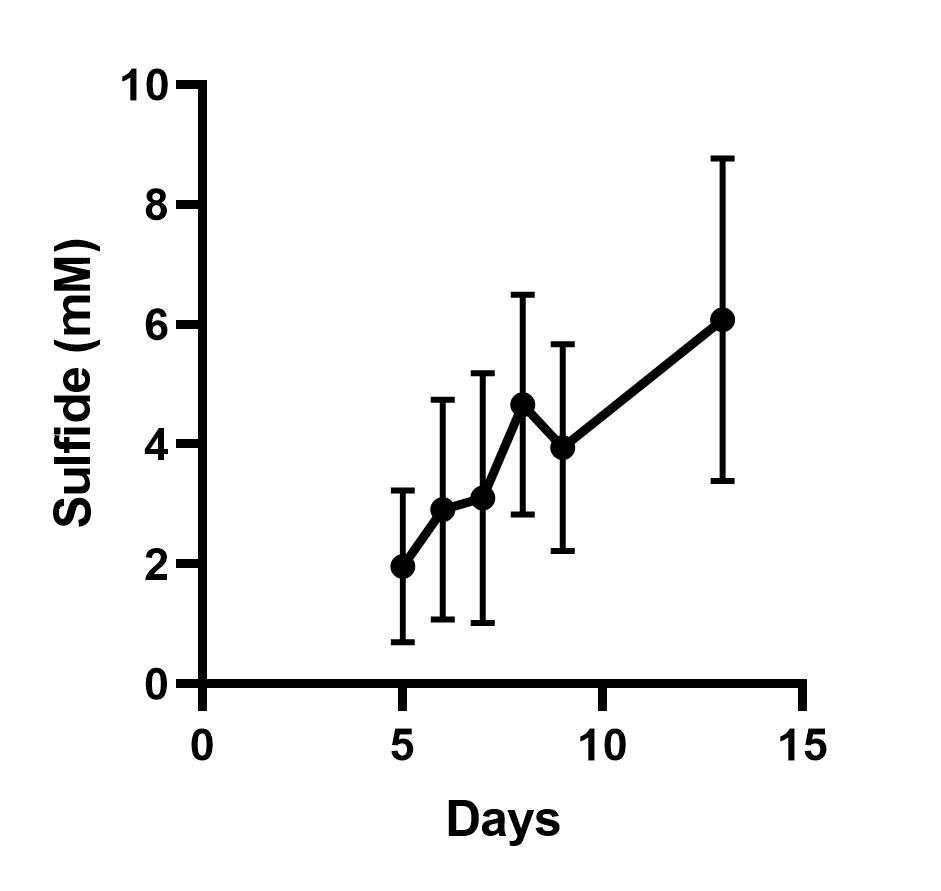


**Supplementary Figure 1.** Average sulfide concentrations from 'equivalent' biofilms (n=3 or n=5) established in all biofilm flow cell experiments. The sulfide data shown are from the flow cell operation phase wherein medium was added to the initially established biofilms prior to treatment with varying concentrations of SNP or ADBAC. **A.** 5 replicate biofilms for experiments treated with 0-150 ppm SNP (Fig. 3A); **B.** 3 replicate biofilms for experiments treated with 0-750 ppm SNP (Fig. 3B); **C.** 3 replicate biofilms for experiments treated with 0-30 ppm SNP (Suppl. Fig. 5); **D.** 3 replicate biofilms for experiments treated with 0-150 ppm SNP (Suppl. Fig. 5); **E.** 5 replicate biofilms for experiments treated with 0-500 ppm ADBAC (Fig. 6A); **F.** 5 replicate biofilms for experiments treated with 0-500 ppm ADBAC (Fig. 6B); **G.** 5 replicate biofilms for experiments treated with 0-500 ppm ADBAC (Suppl. Fig. 7); **H.** 5 replicate biofilms for experiments treated with 0-500 ppm ADBAC (Suppl. Fig. 8). Error bars indicate one standard deviation of the mean (n=3 or n=5).

**
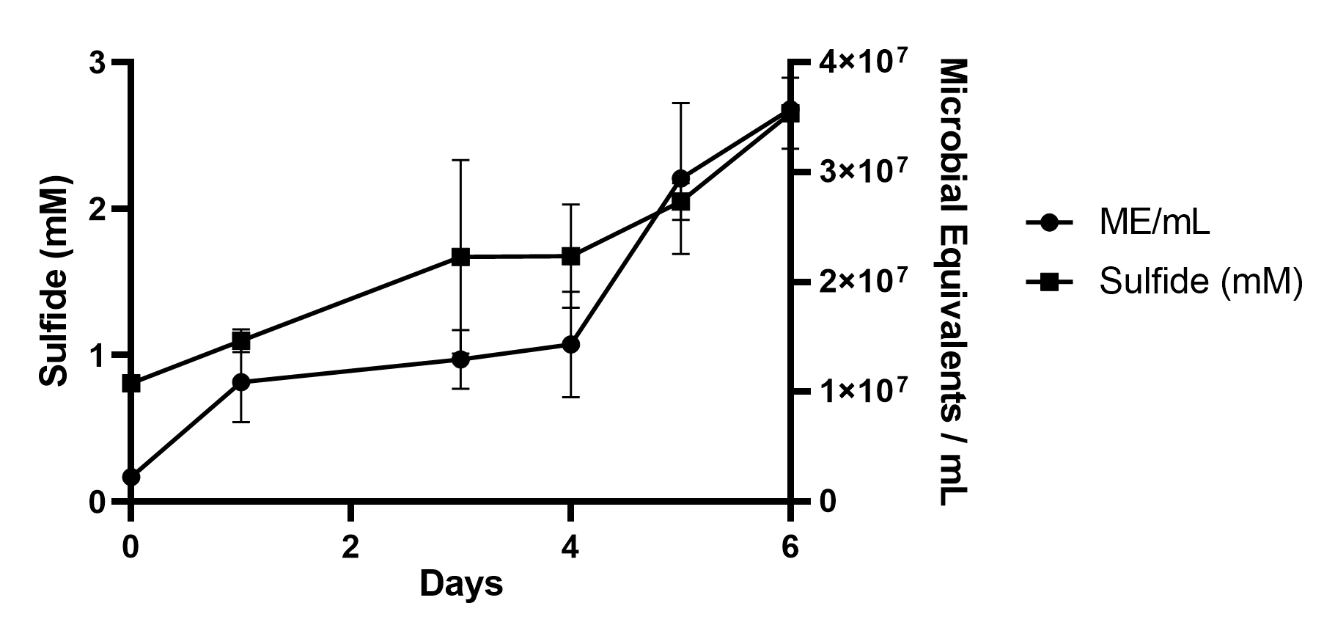
**

**Supplementary Figure 2:** Growth of the planktonic SRM culture as measured by sulfide production and increase in numbers of active cells (as measured using an ATP assay) used to inoculate the biofilm flow cell experiments described in this study. Error bars indicate one standard deviation of the mean (n=3).

**
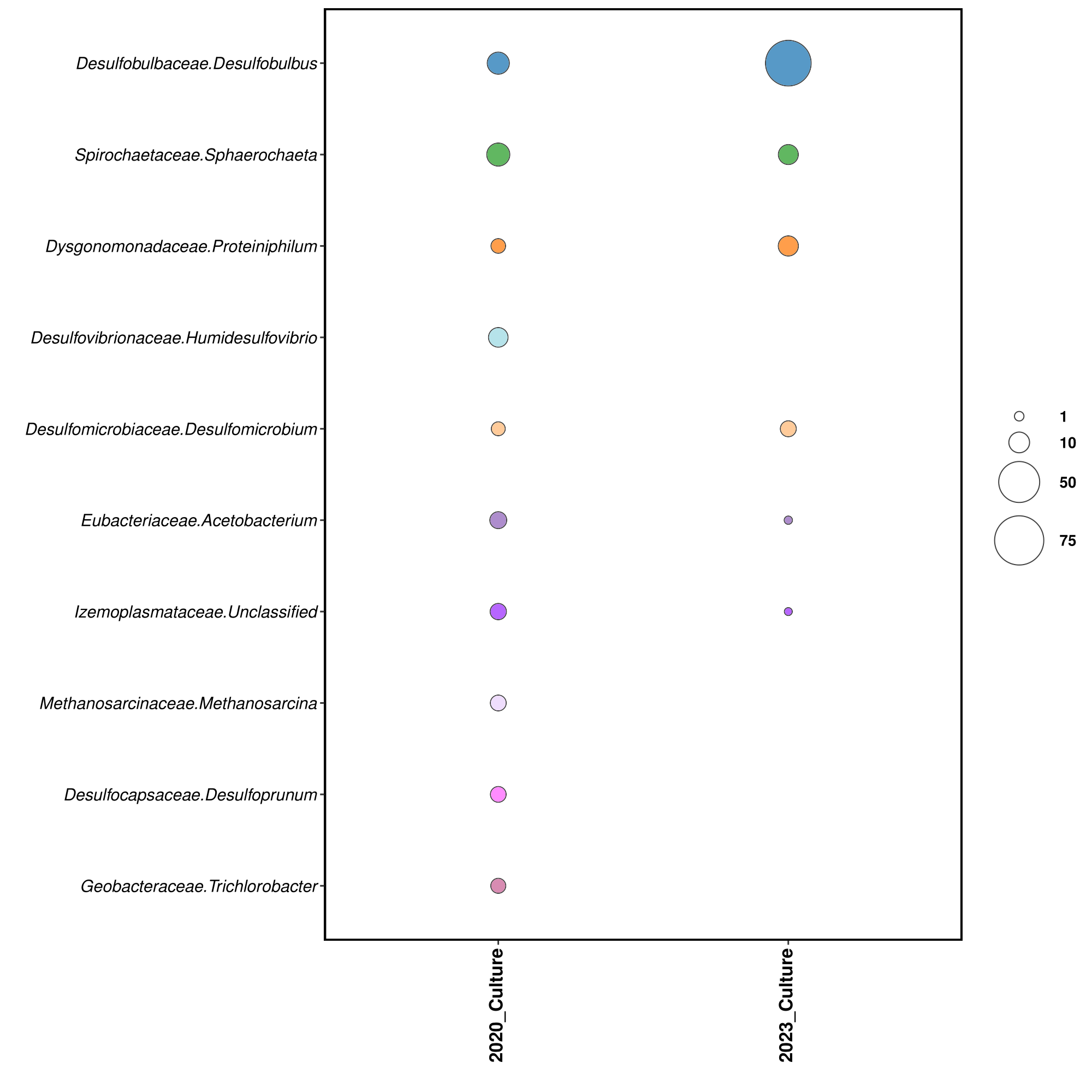
**

**Supplementary Figure 3.** Microbial community composition of the planktonic SRM culture used in this study as sequenced using 16S rRNA gene sequencing in 2020 and in 2023. The top 10 most abundant (e.g., highest relative abundance) ASVs are shown. The R scripts used for data processing and visualization can be found at https://github.com/kiragoff.

**

**

**

**

**Supplementary Figure 4.** Effects of various concentrations of SNP on sulfide production (top) and sulfate consumption (bottom) by the planktonic SRM culture used in this study. Error bars indicate one standard deviation of the mean for each tested condition (n=3) for the sulfide results. Sulfate analysis was only conducted from one replicate bottle of each treatment to confirm the sulfide results.


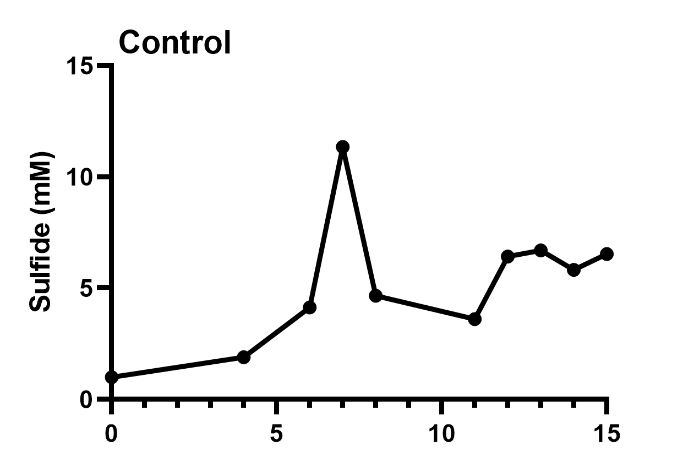

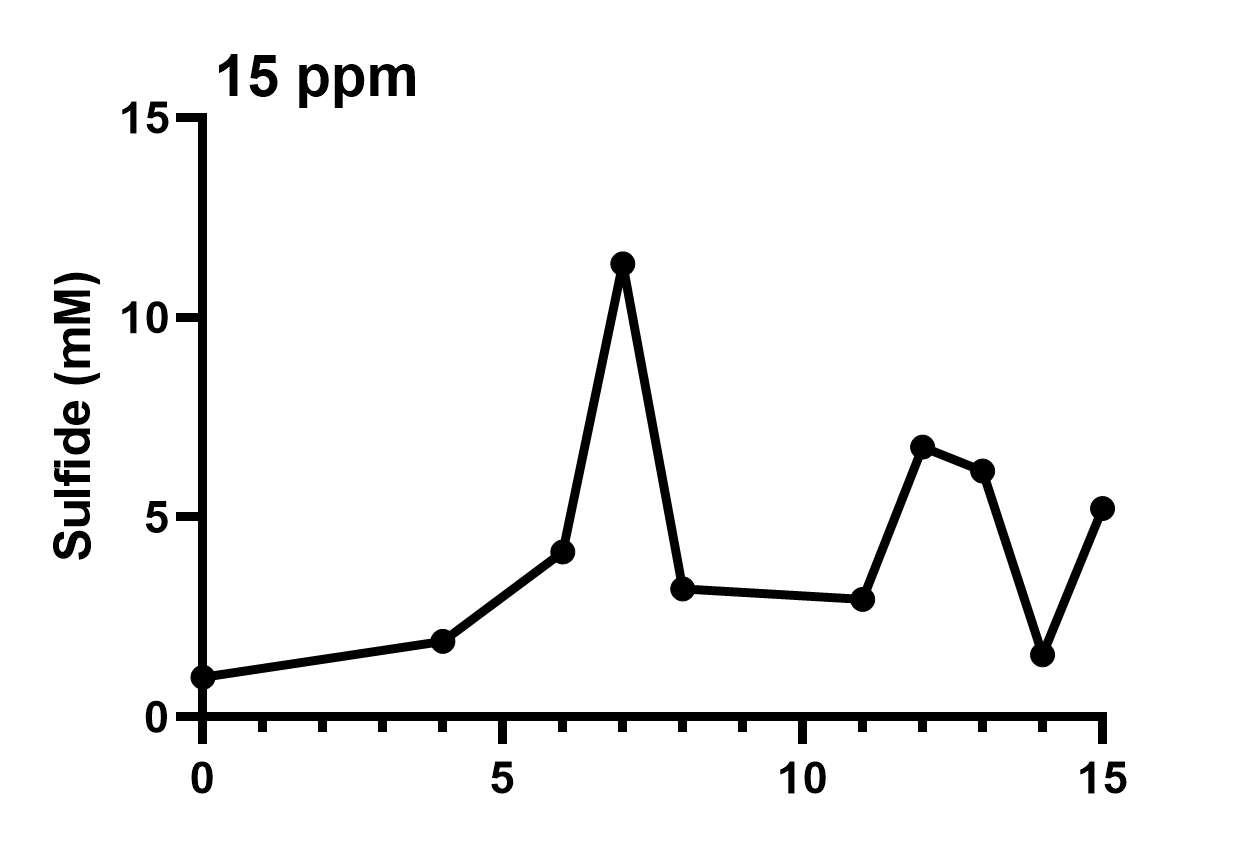

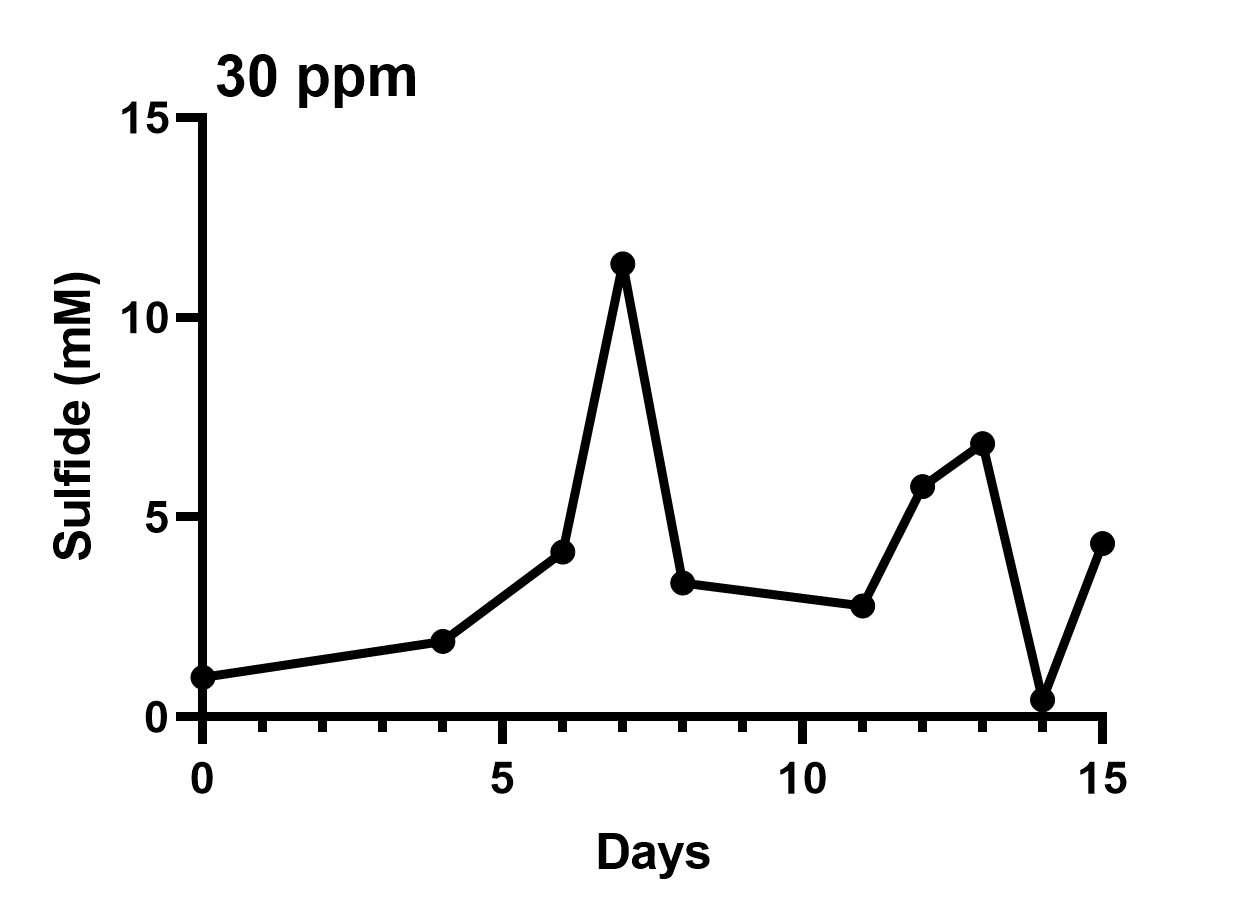

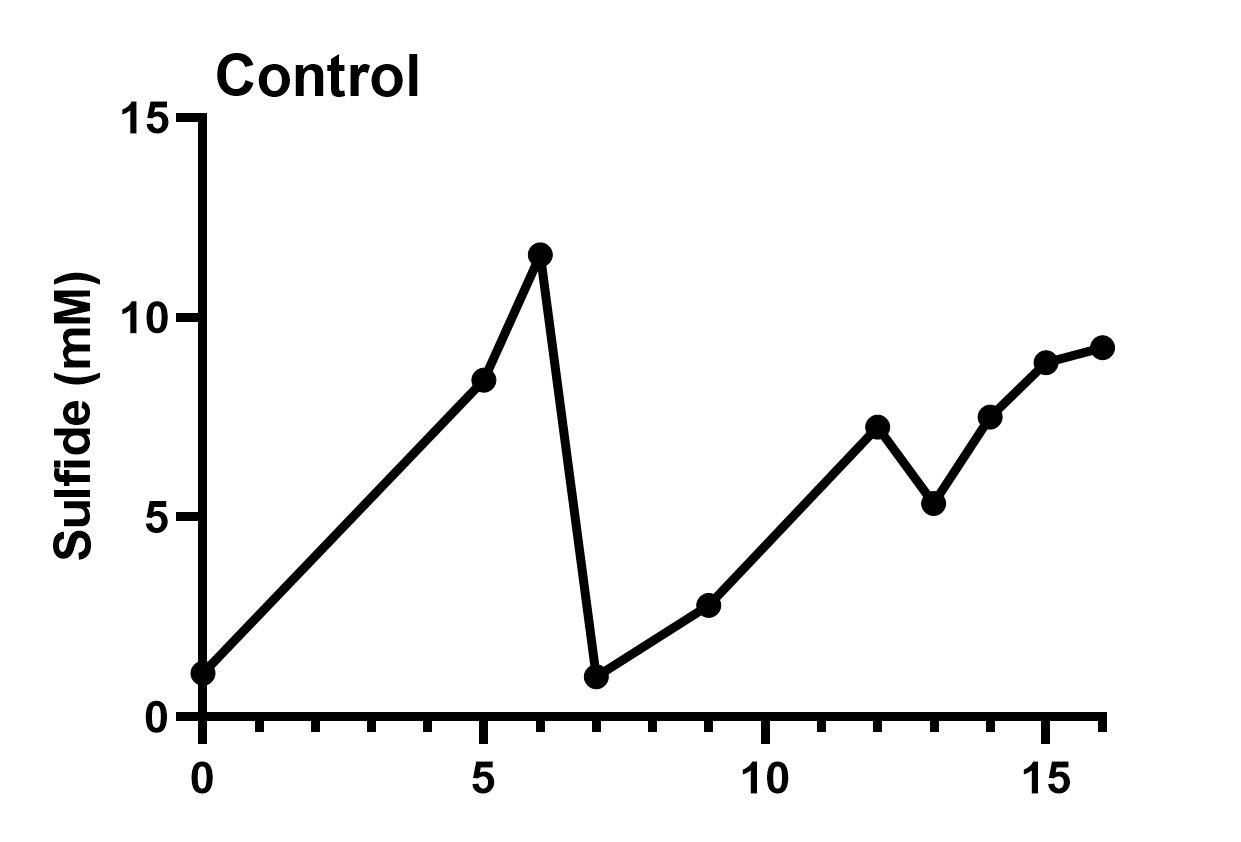

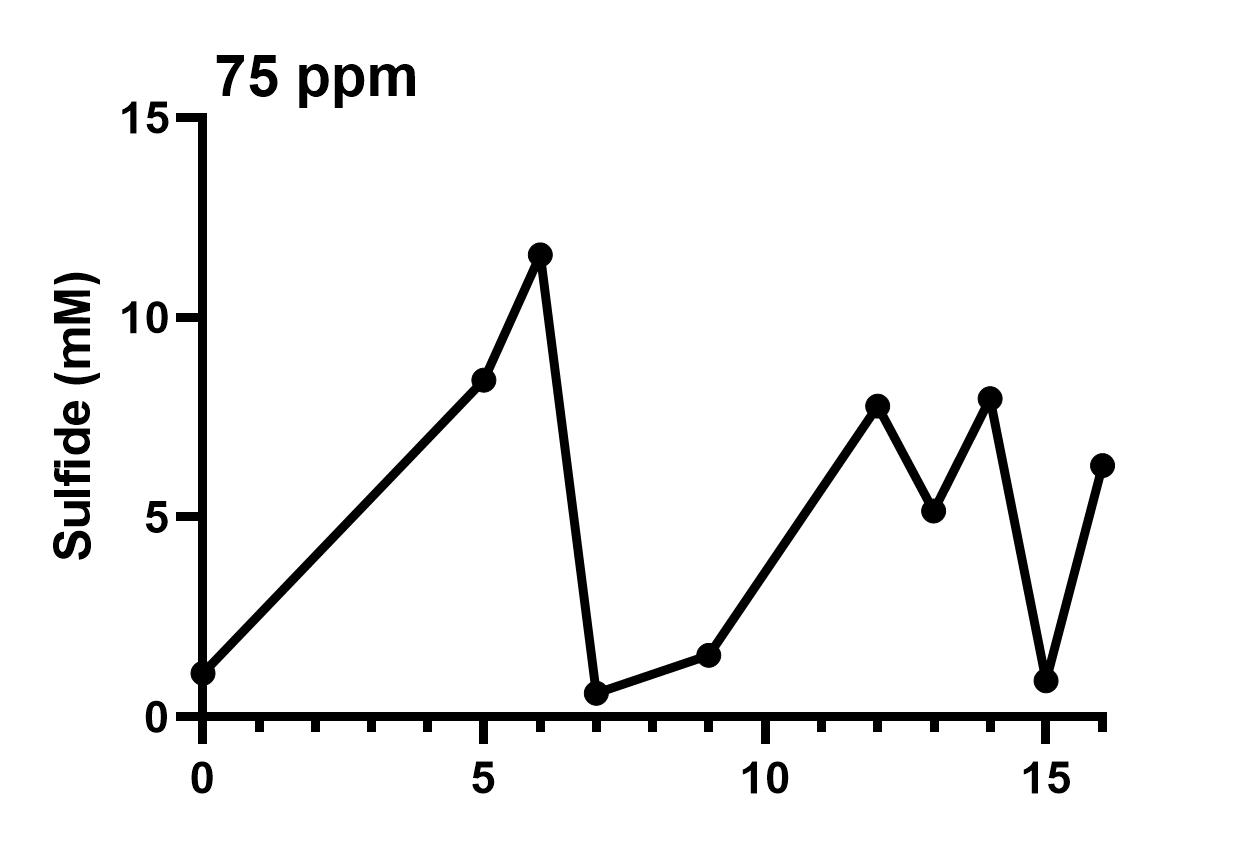

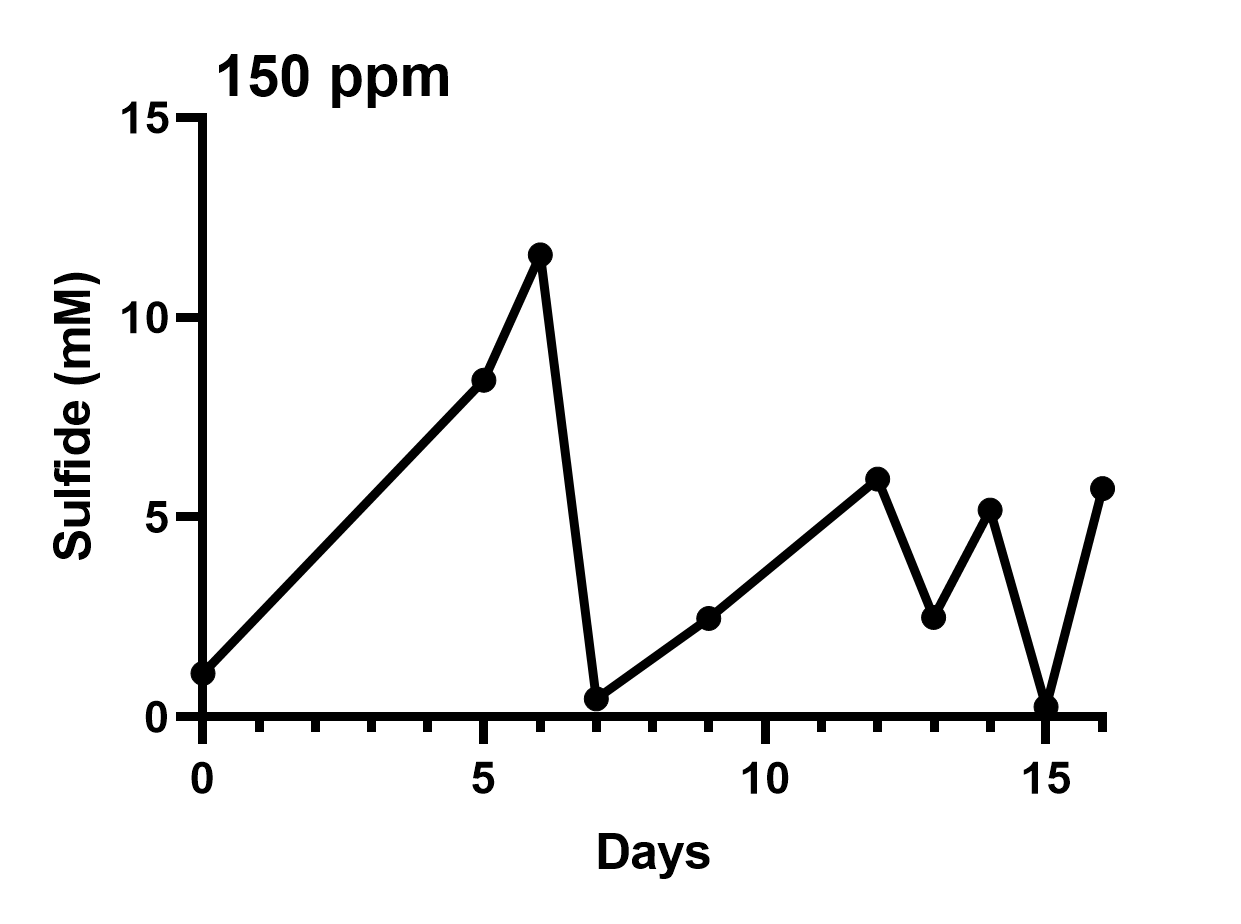


**Supplementary Figure 5.** Initial biofilm flow cell experiments testing the effects of 15 or 30 ppm SNP (left) or 75 or 150 ppm SNP (right) on existing SRM biofilms. These experiments were conducted separately and are replicates of the data shown in Figure 3A. The blue dotted lines indicate the time of medium flow into the biofilms, and the red dotted line indicates the time of biocide treatment.

**

**

**Supplementary Figure 6.** Effects of various concentrations of ADBAC on sulfide production by the planktonic SRM culture used in this study. The arrow indicates the time point at which cultures were transferred to fresh medium to determine cell viability. Error bars indicate one standard deviation of the mean for each tested condition (n=3).


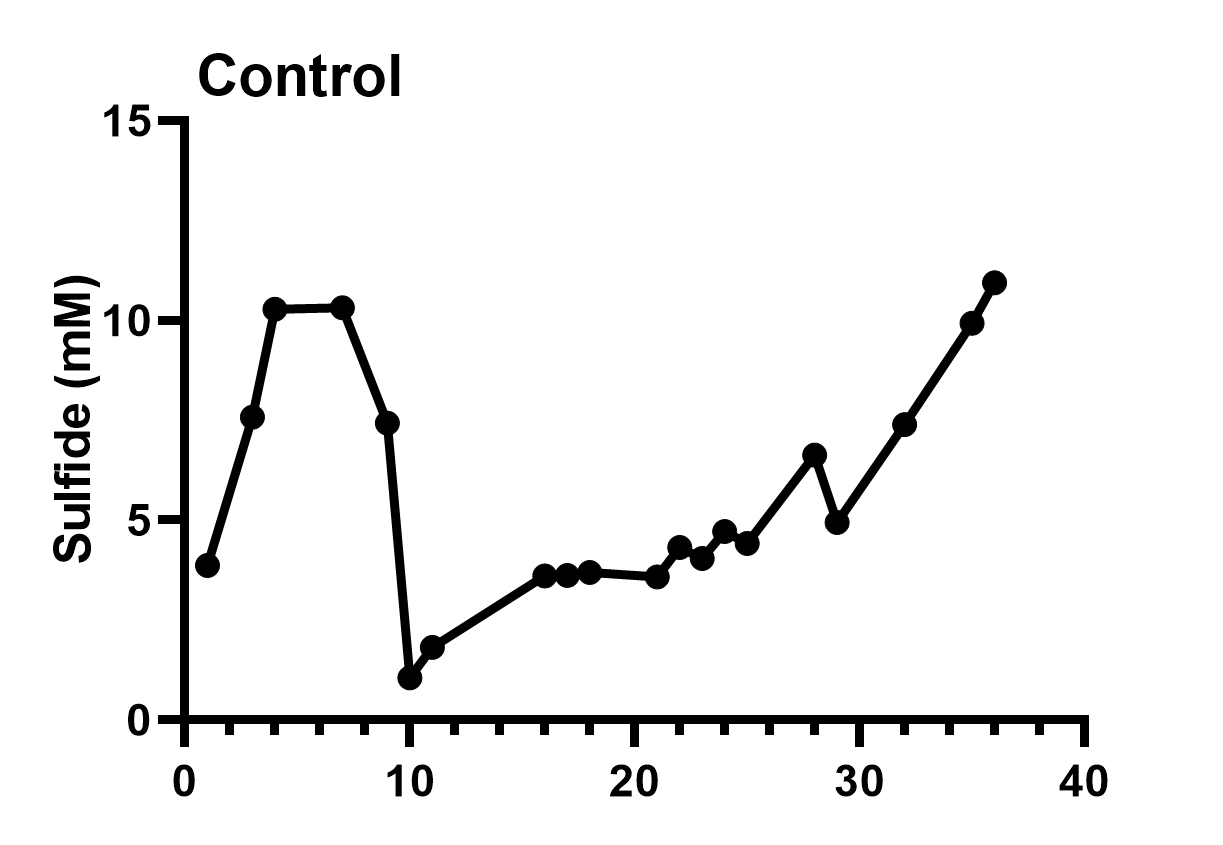

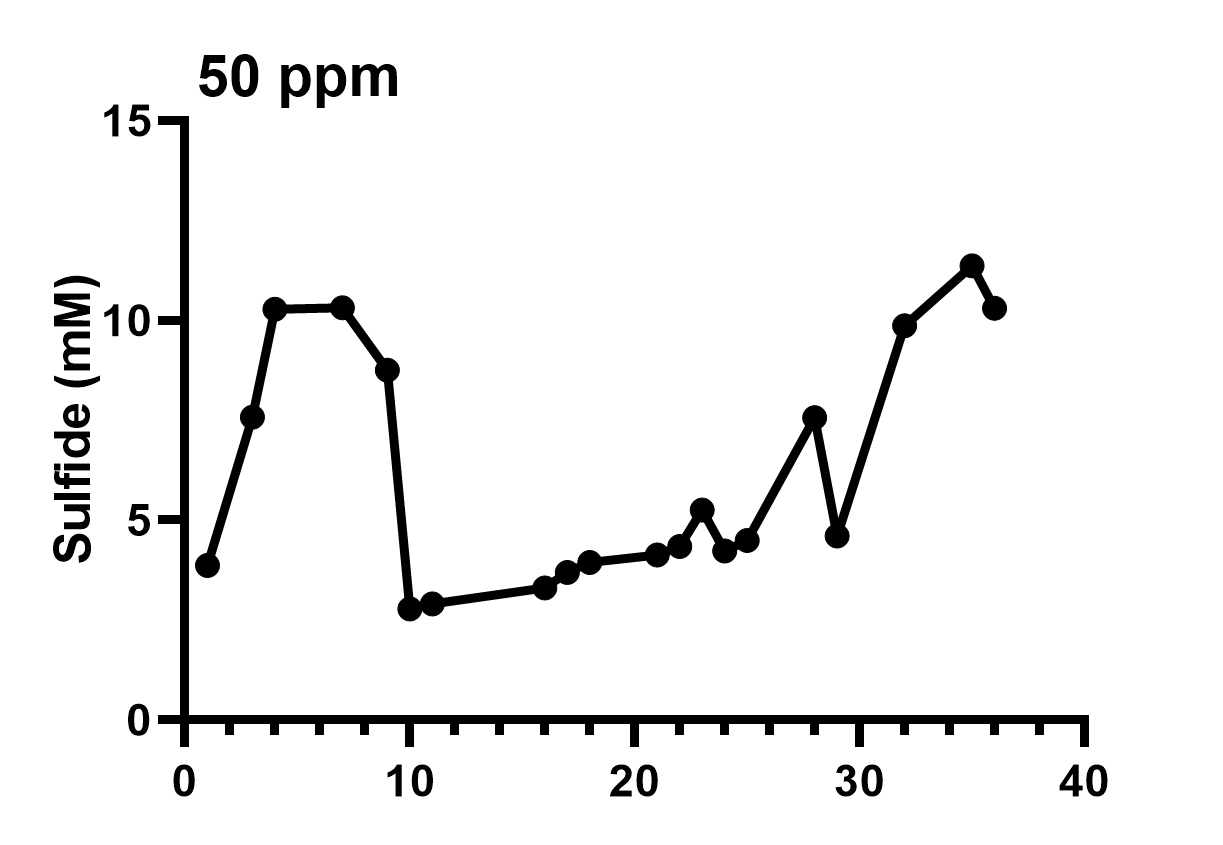

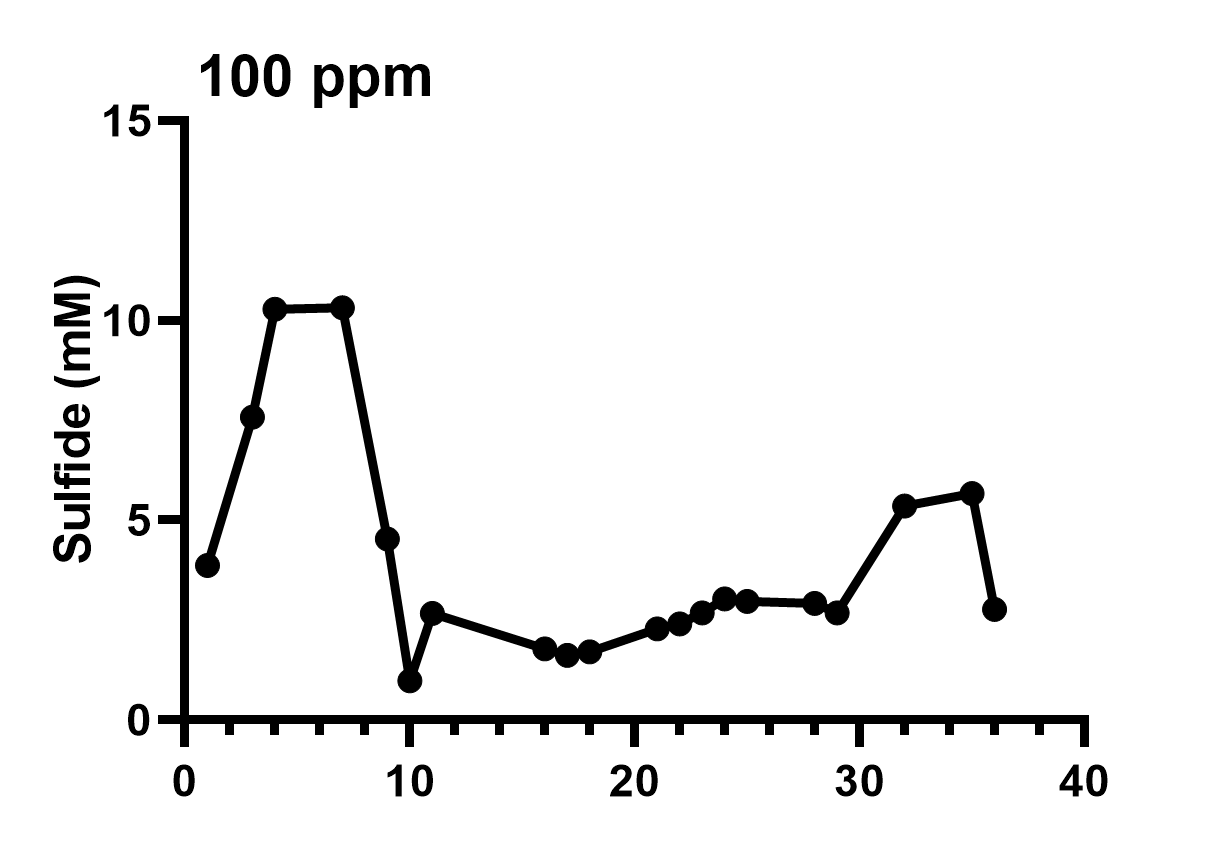

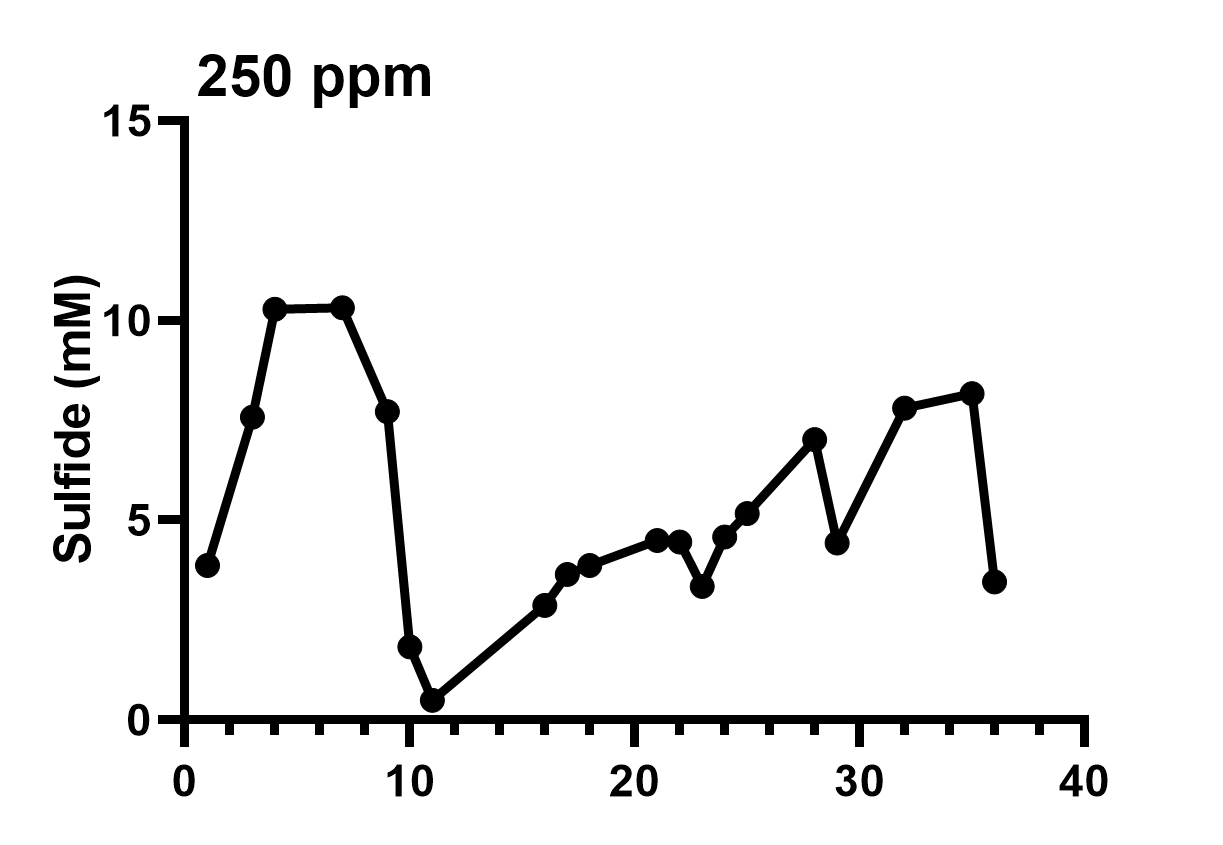

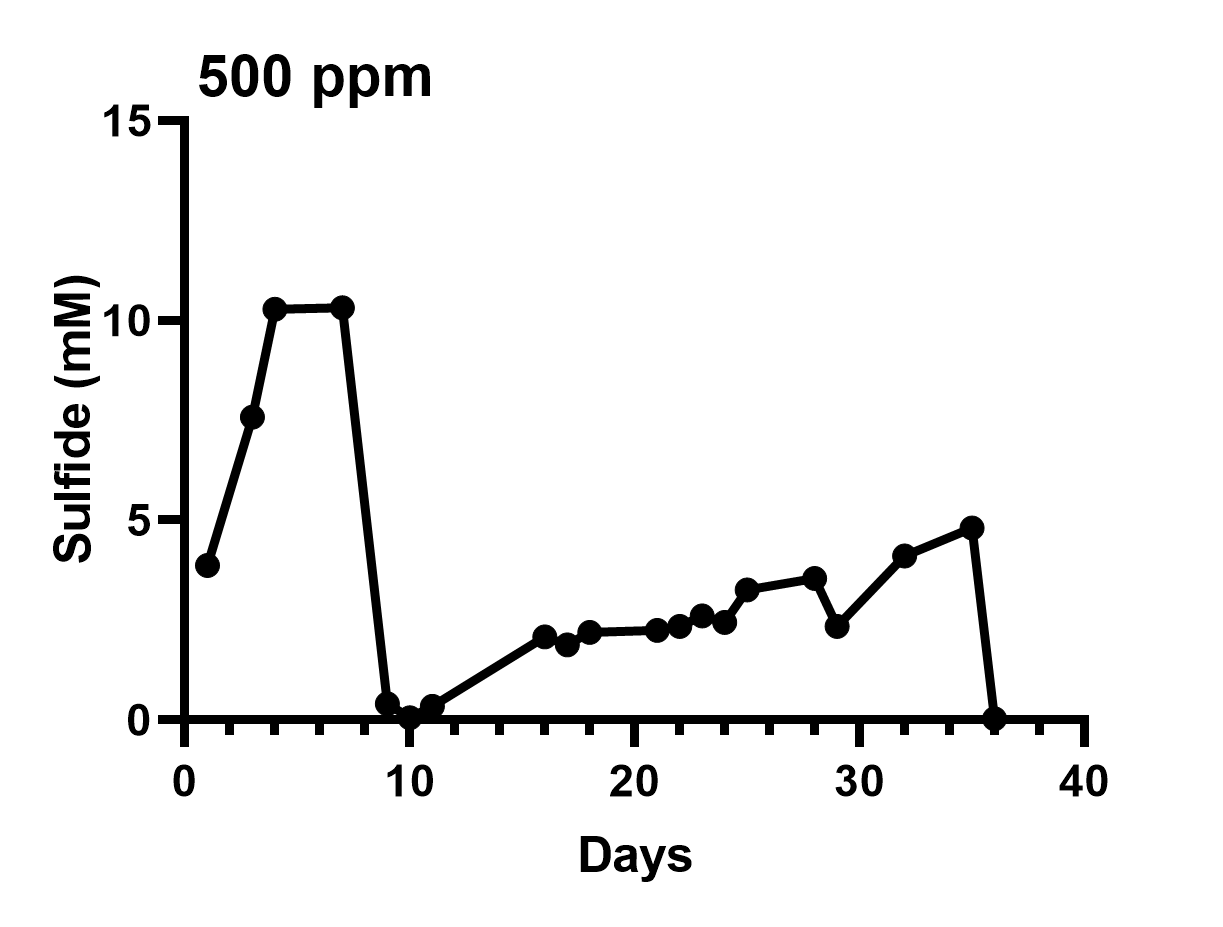


**Supplementary Figure 7.** Repeated biofilm flow cell experiment wherein biofilms were treated with varying concentrations of ADBAC for 14 h prior to their preparation for two-photon microscopy. The blue dotted lines indicate the time of medium flow into the biofilms, and the red dotted line indicates the time of biocide treatment. This experiment is a replicate of that shown in Figure 6A wherein the flow cells were dismantled shortly after biocide treatment.


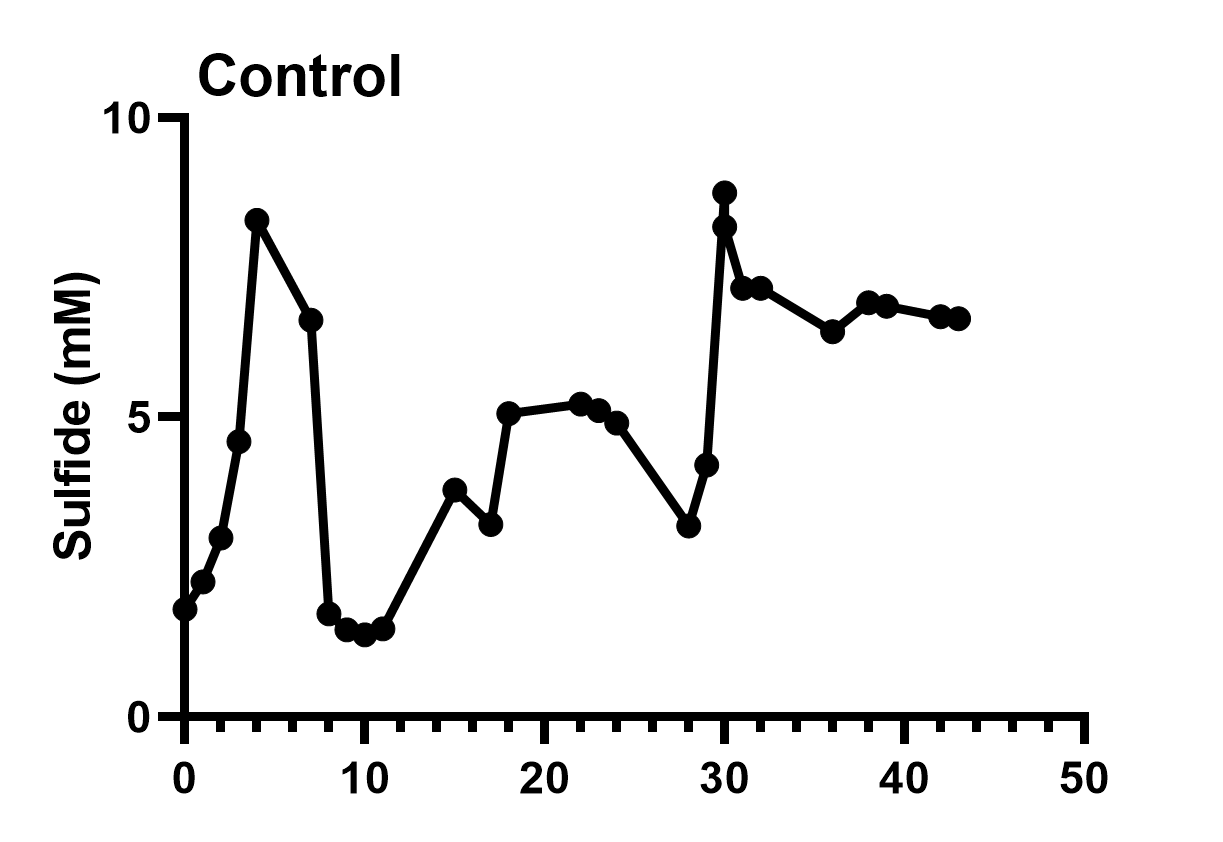

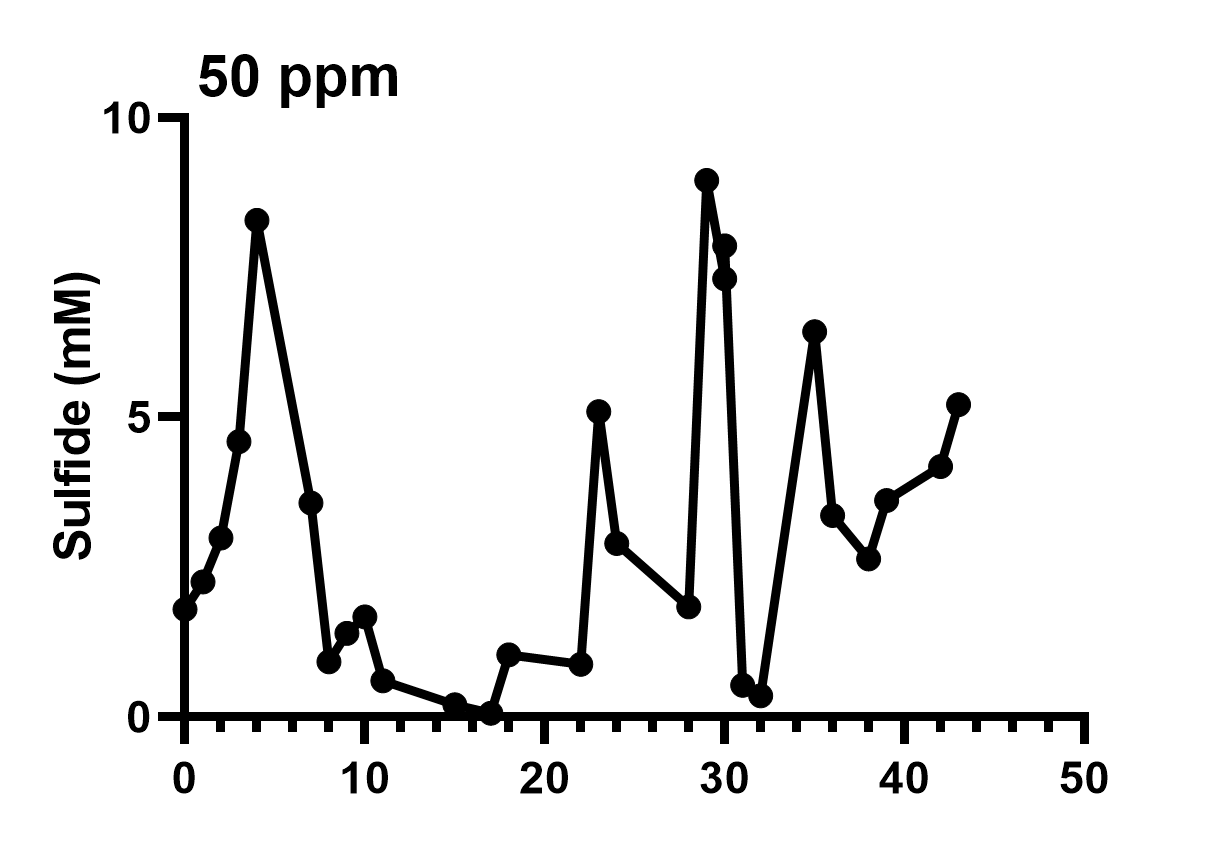

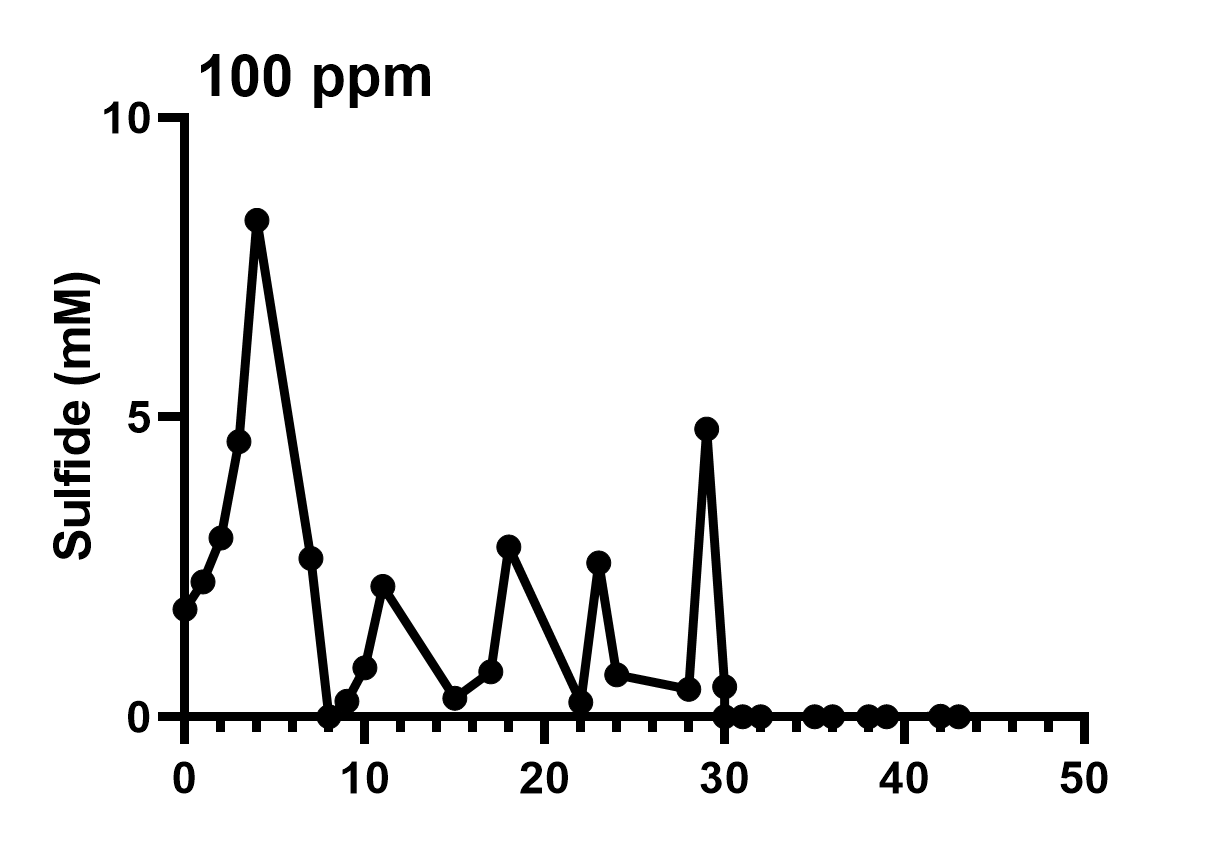

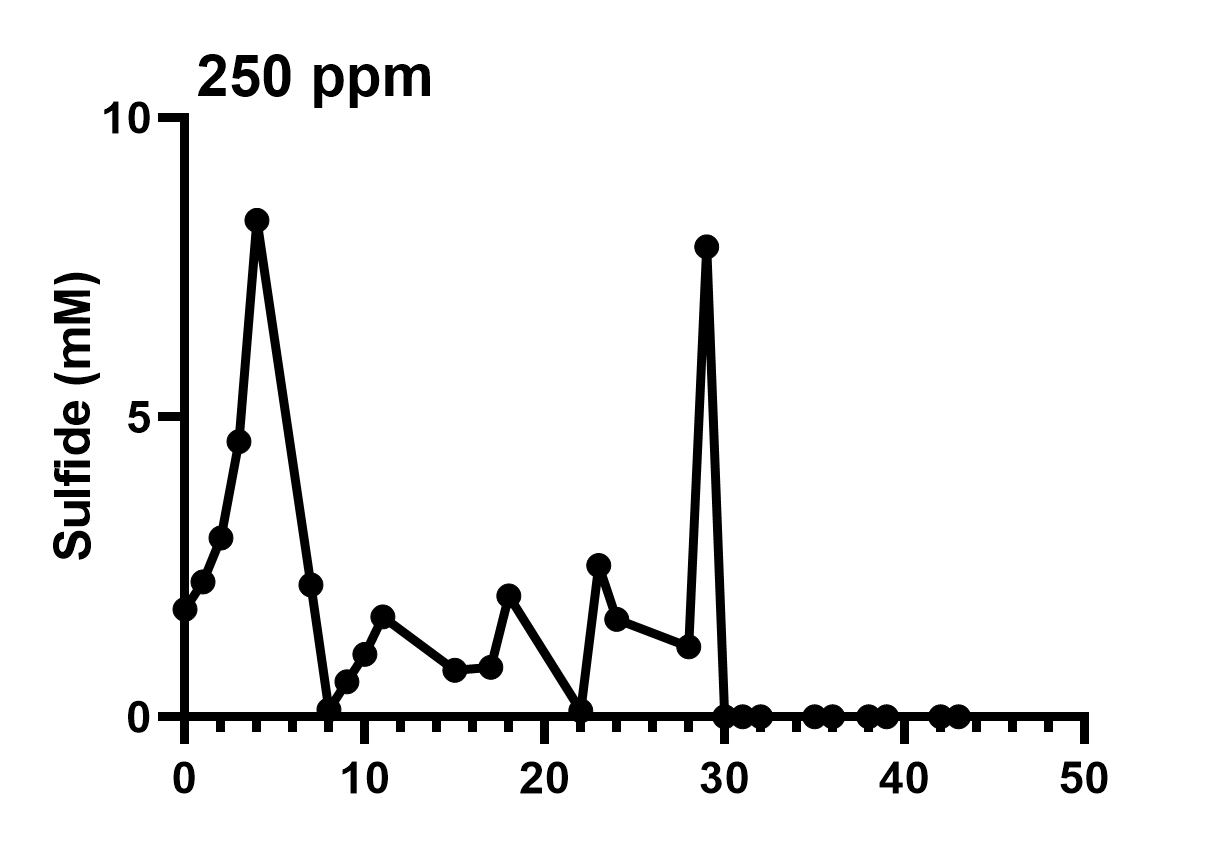

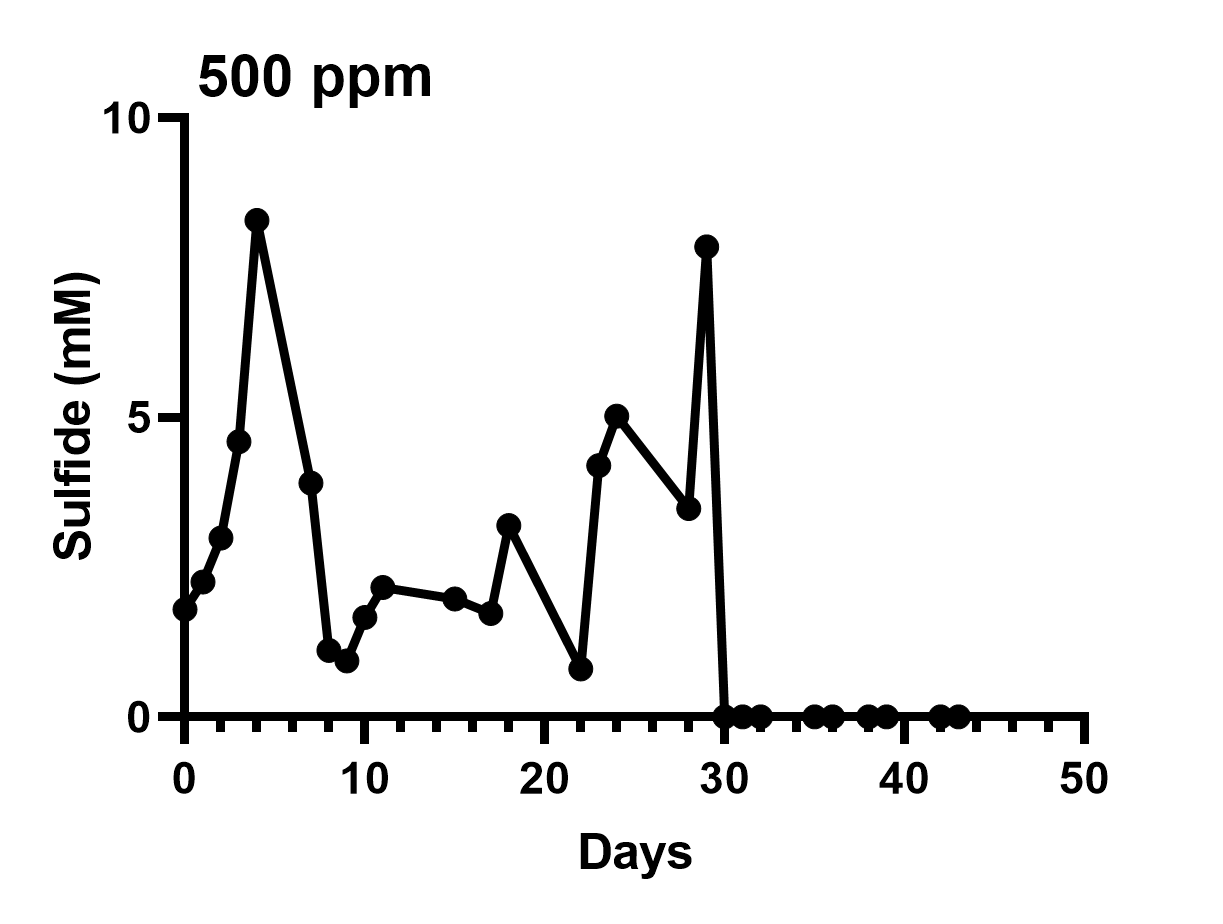


**Supplementary Figure 8.** Repeated biofilm flow cell experiment testing the effects of sulfide resumption following the treatment of sulfate-reducing biofilms with varying concentrations of ADBAC. The blue dotted lines indicate the time of medium flow into the biofilms, and the red dotted line indicates the time of biocide treatment. This experiment was conducted as a replicate of the experiment shown in Figure 6B wherein the medium was added to the flow cells following biocide treatment to determine whether sulfide production would resume.
